# Supplementary material for: Hyperosmotic Stress Induces Phosphorylation of CERT and Enhances Its Tethering throughout the Endoplasmic Reticulum
Source: Int J Mol Sci. 2022 Apr 5;23(7):4025. doi: 10.3390/ijms23074025 (PMC8999913; doi:10.3390/ijms23074025)

Figure S1A

WB: CERT

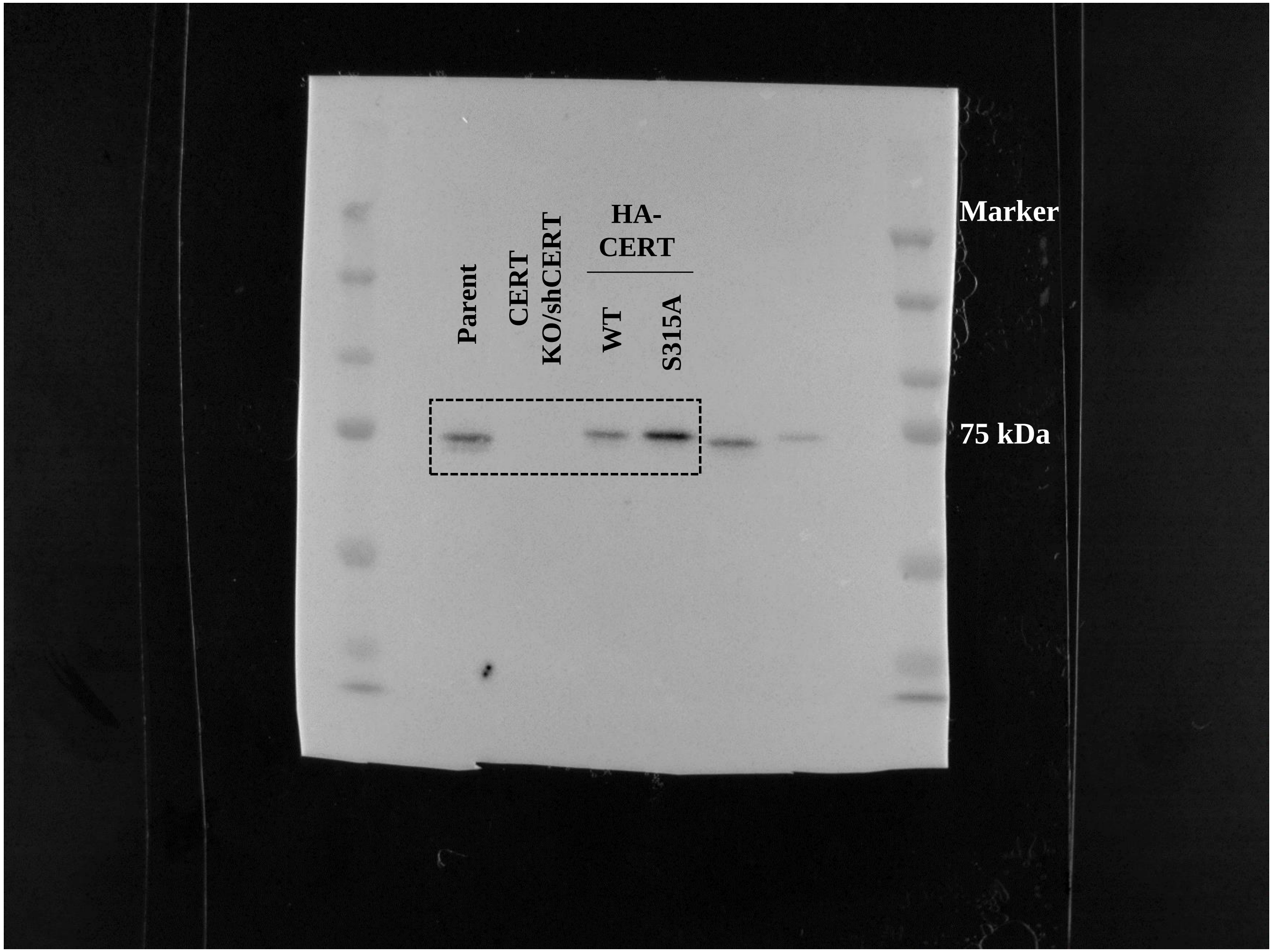

Figure S1A

WB: HA

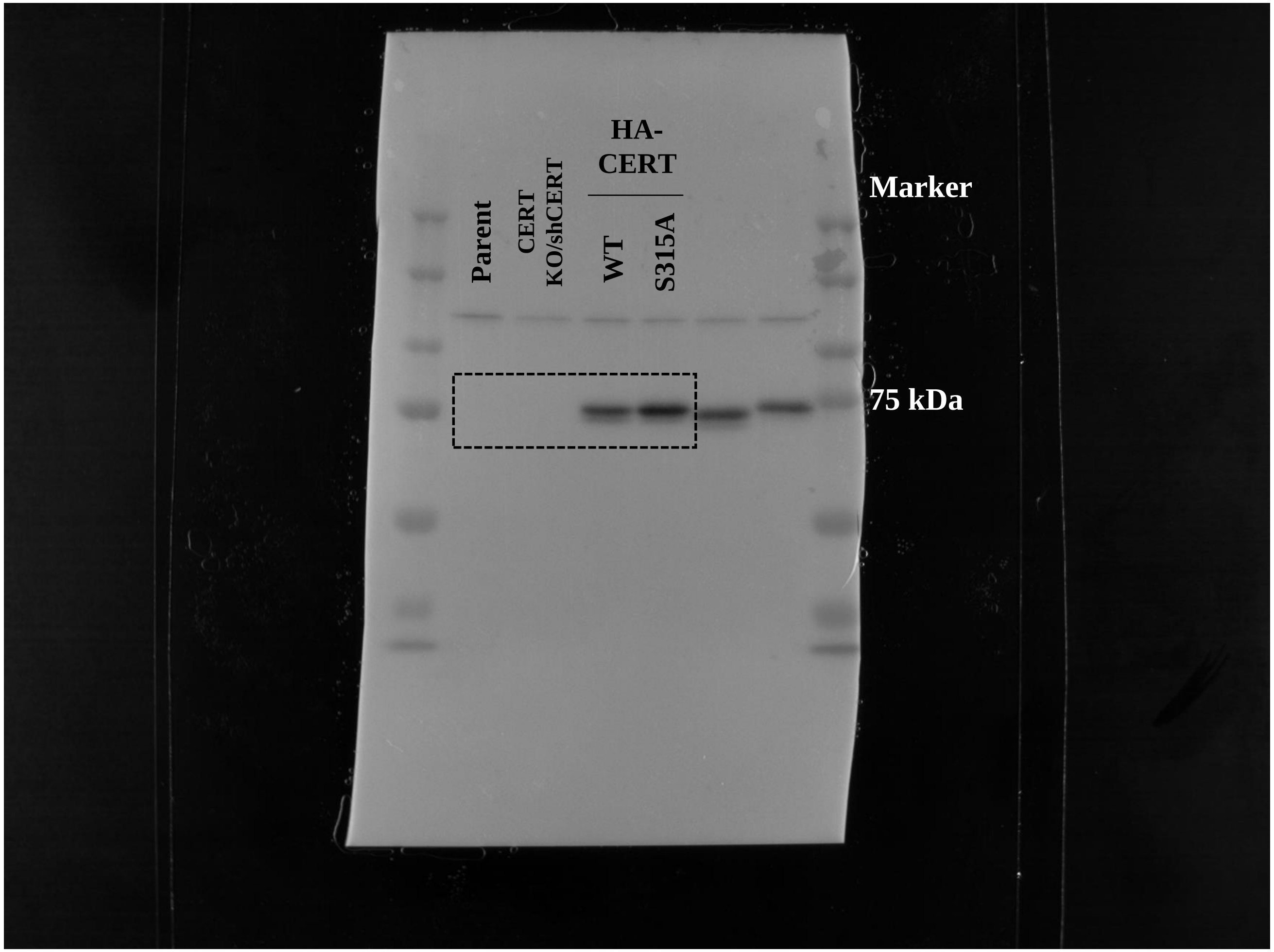

Figure S1A

WB: GAPDH

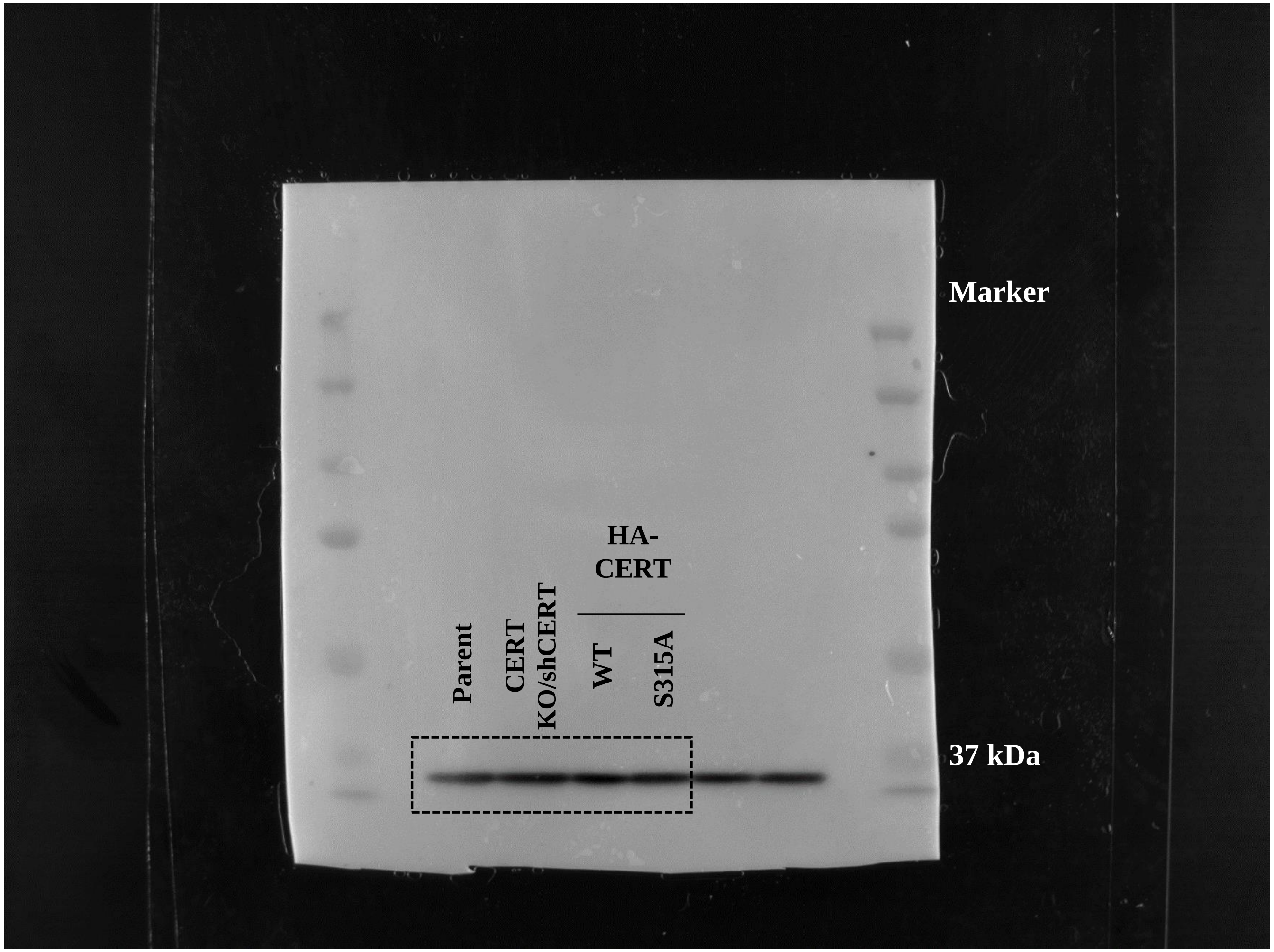

Figure S1B

IP: HA_WB: pS315

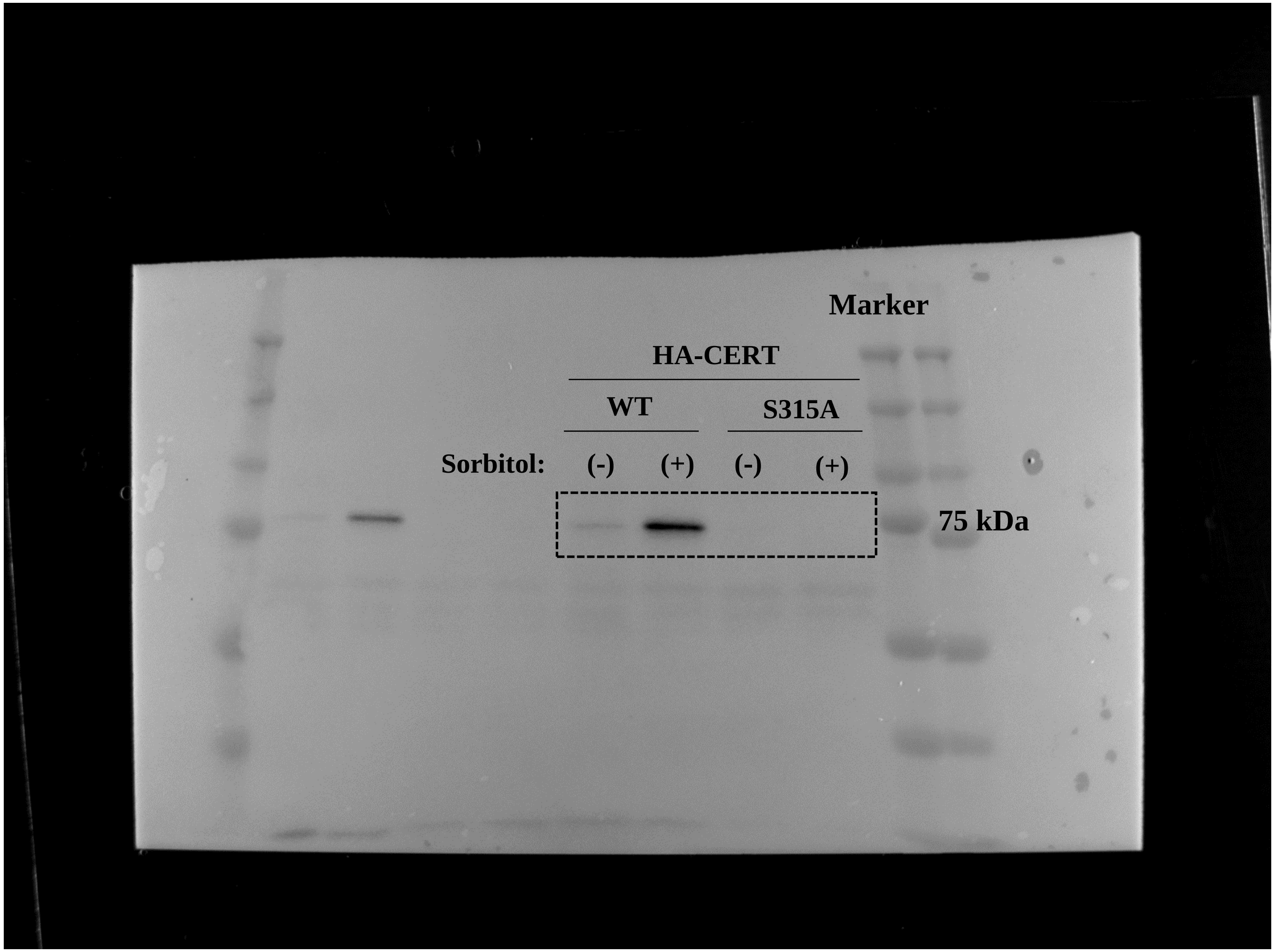

Figure S1B

IP: HA_WB: HA

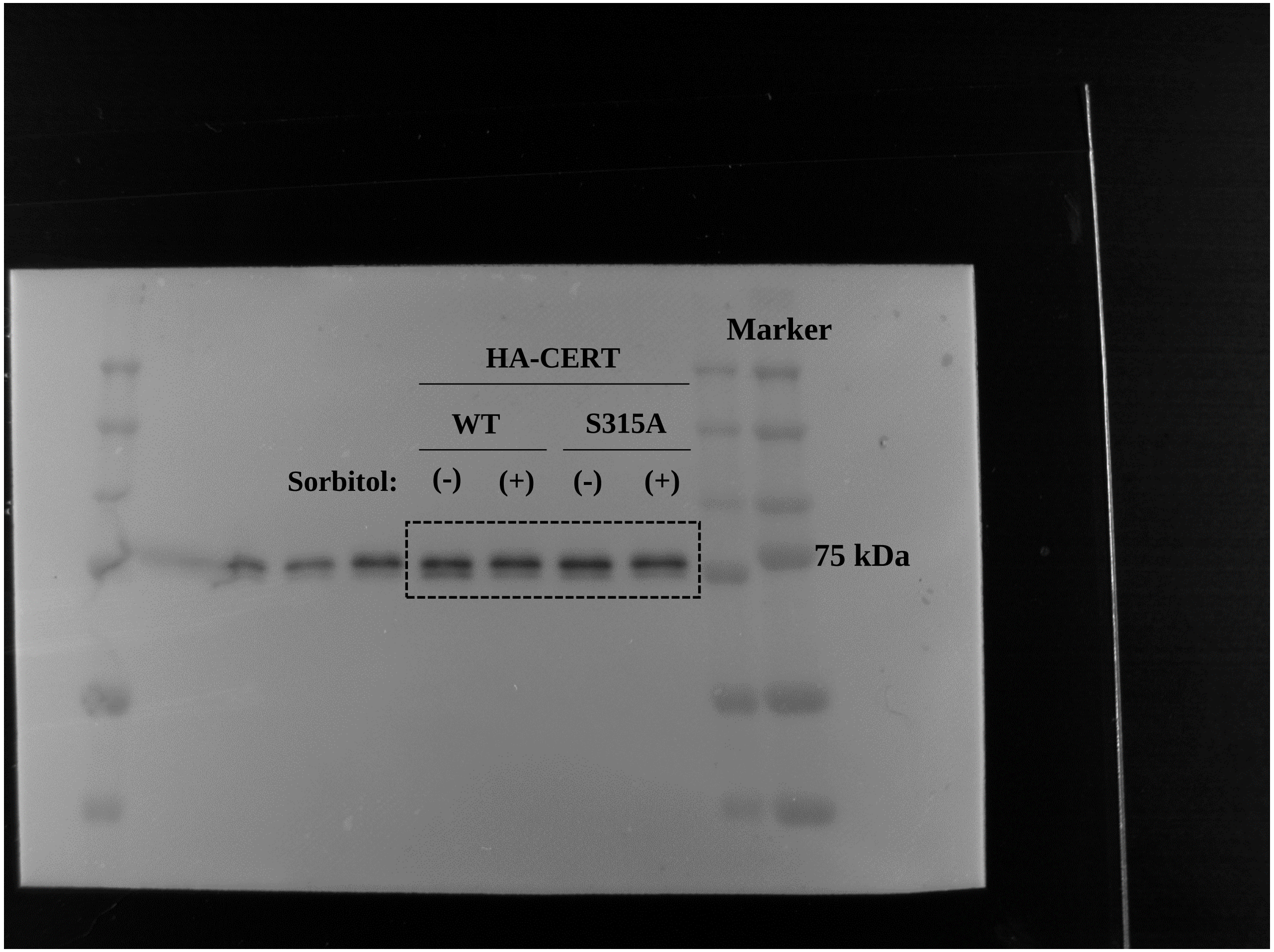

Figure S1C

WB: CERT

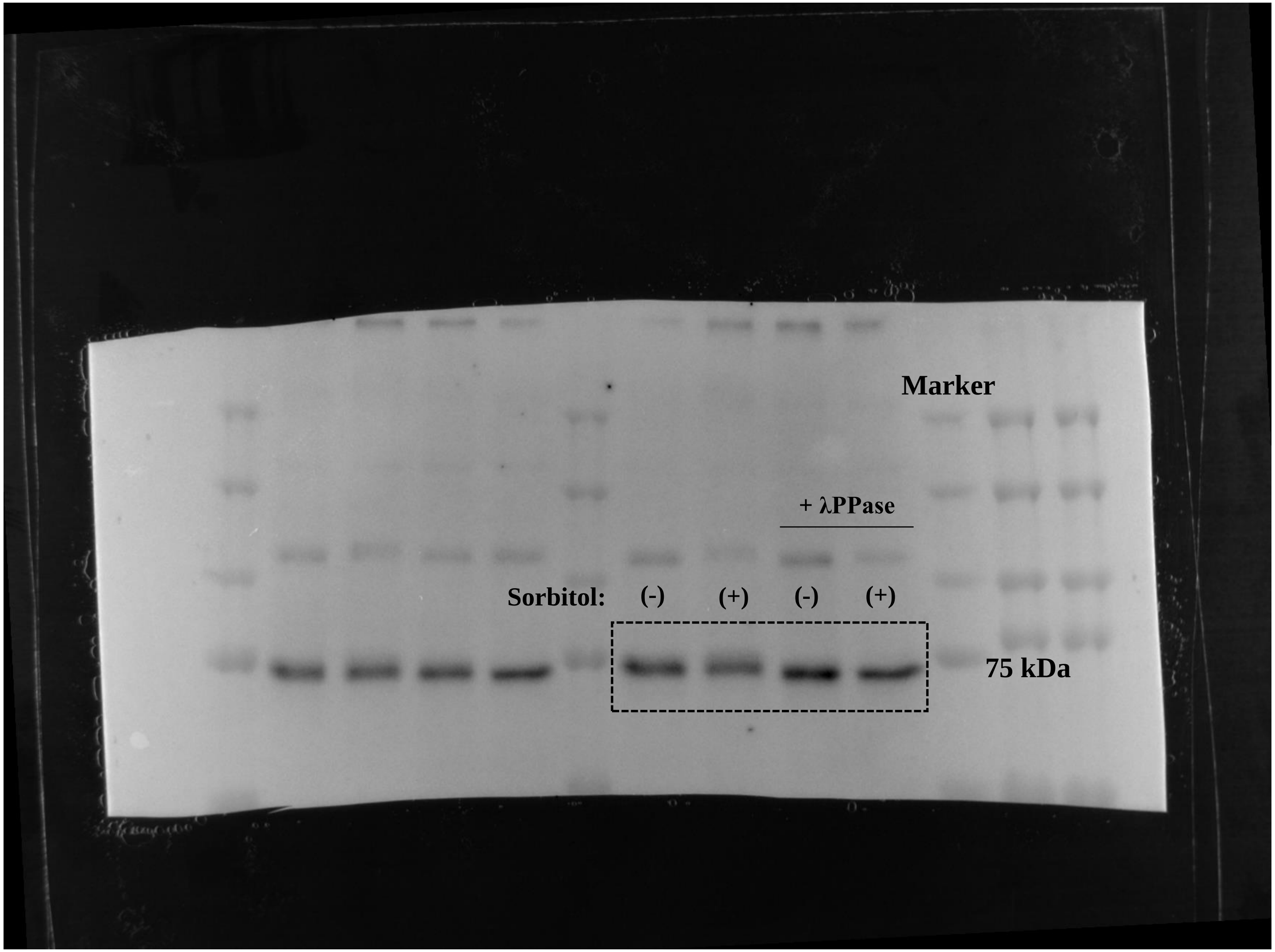

Figure S1C

WB: HA

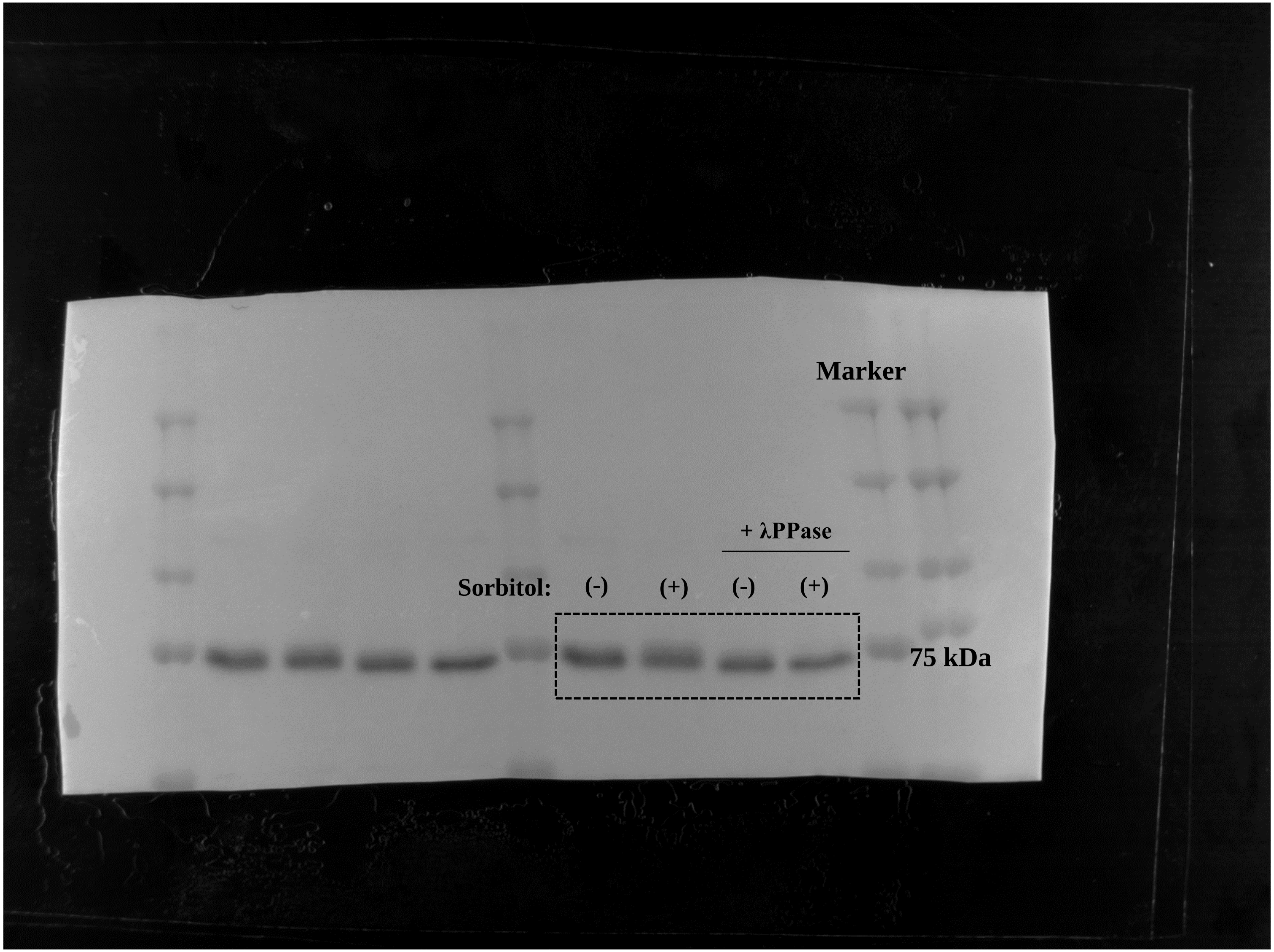

Figure S1C

WB: GAPDH

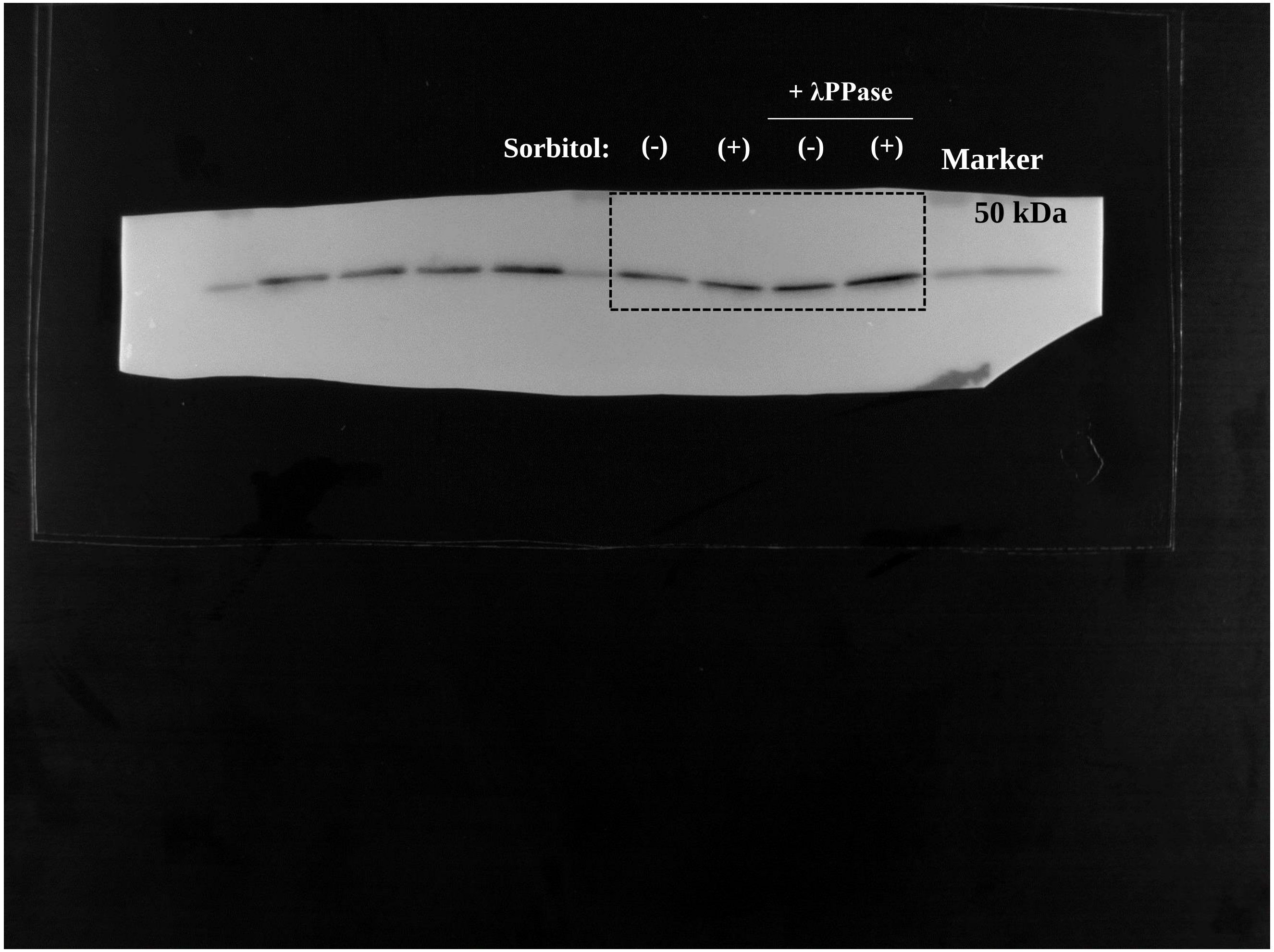

Figure S1D

IP: HA_WB: pS315

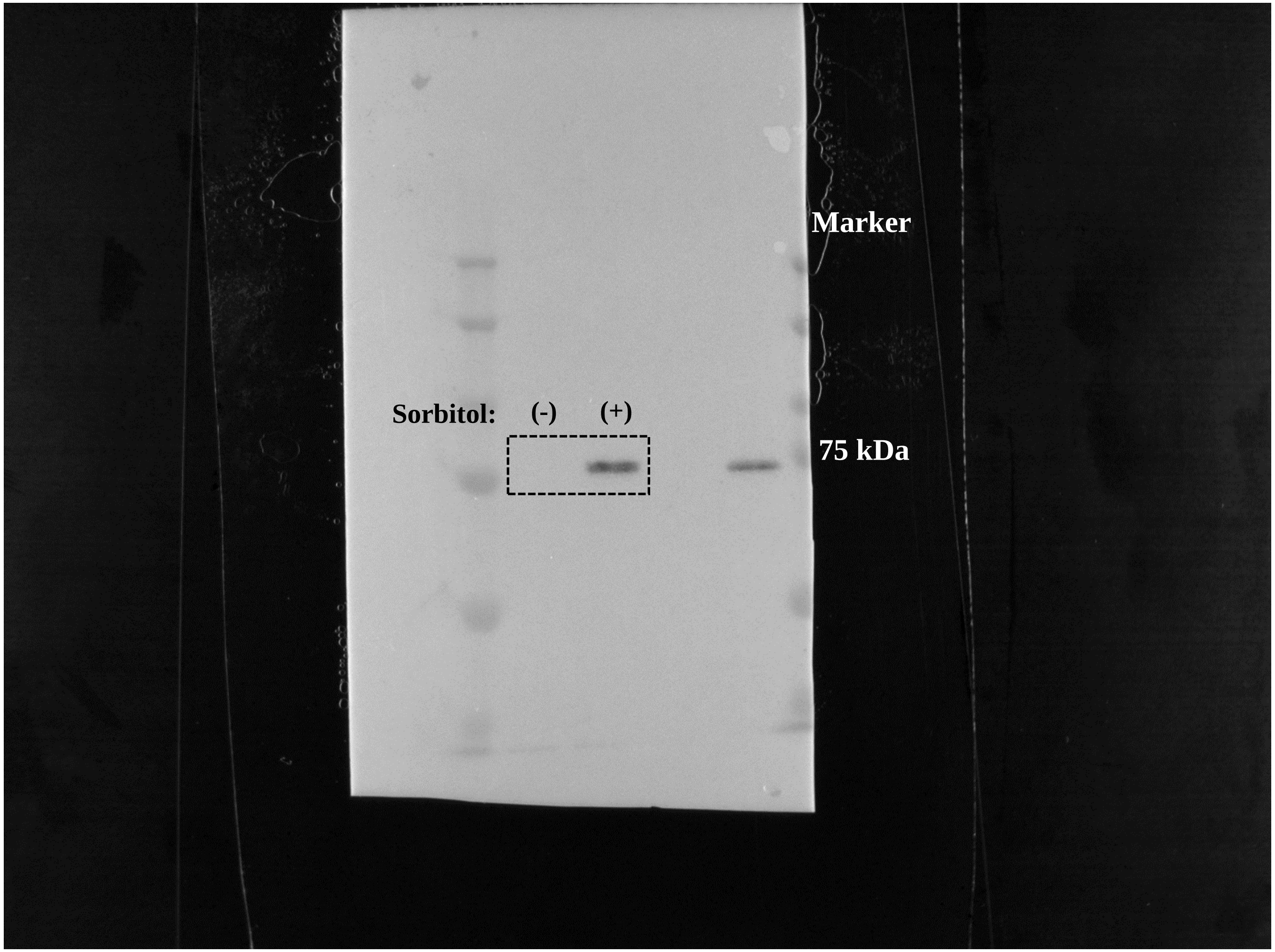

Figure S1D

IP: HA_WB: HA

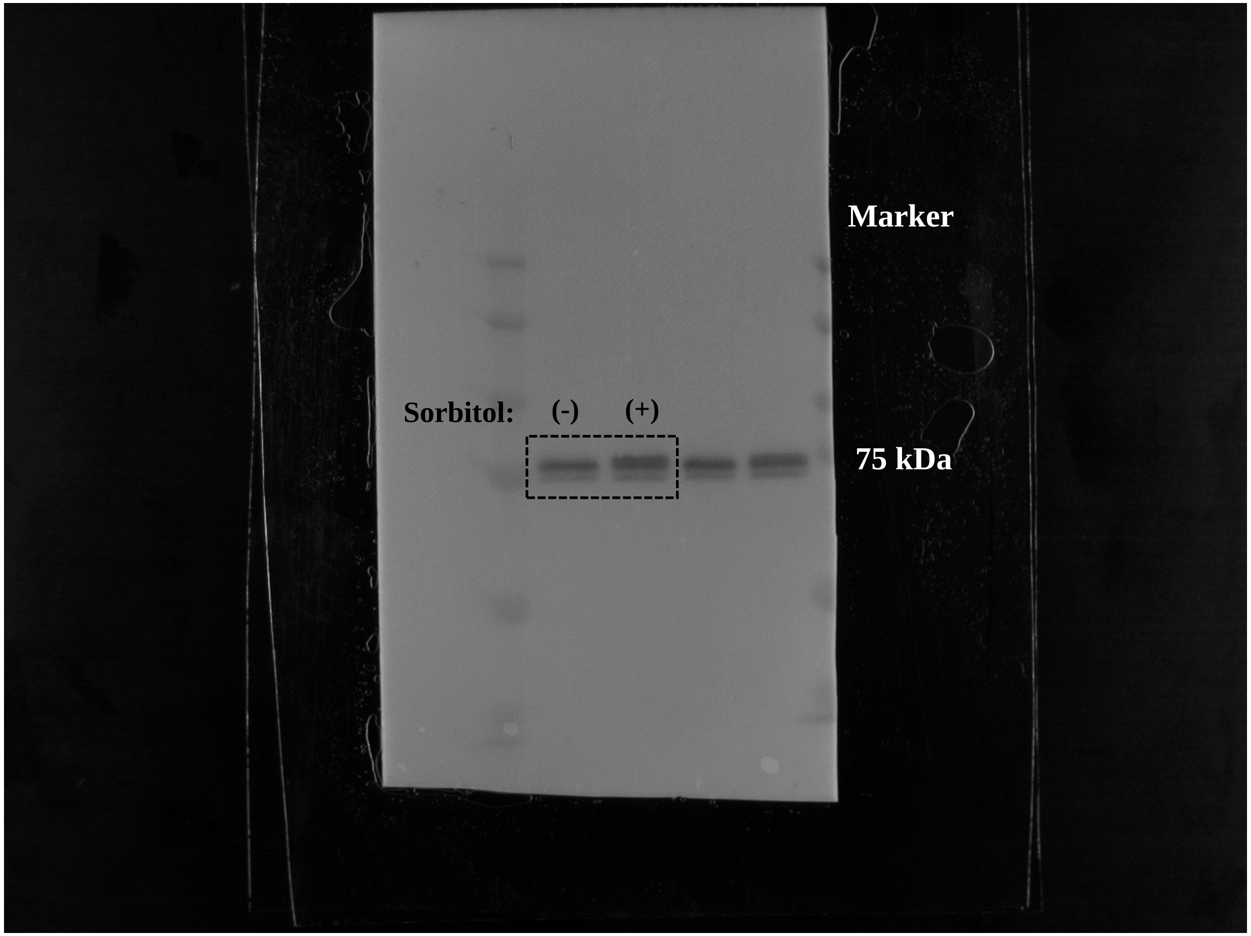

Figure S1D

WB: pS315

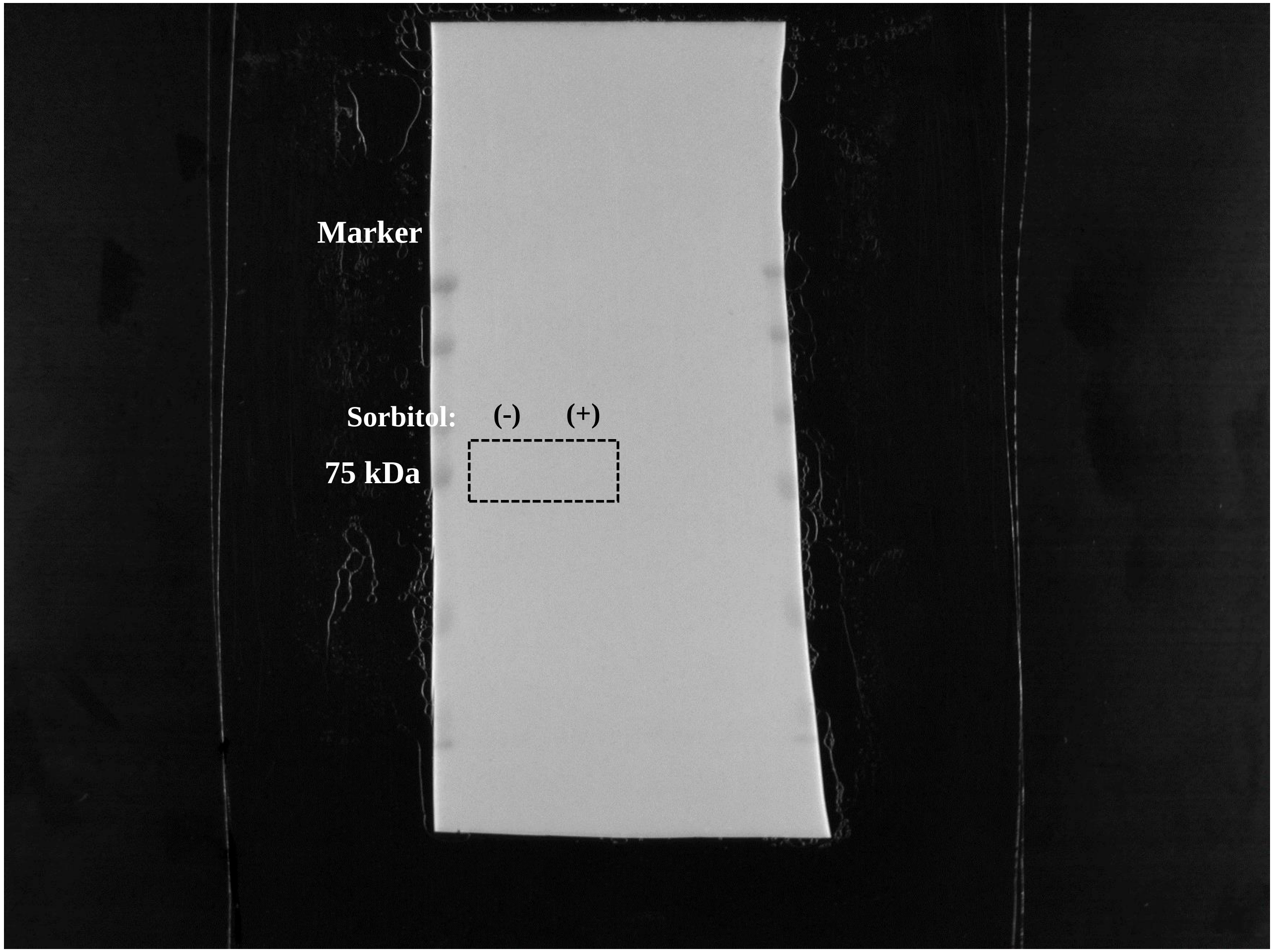

Figure S1D

WB: HA

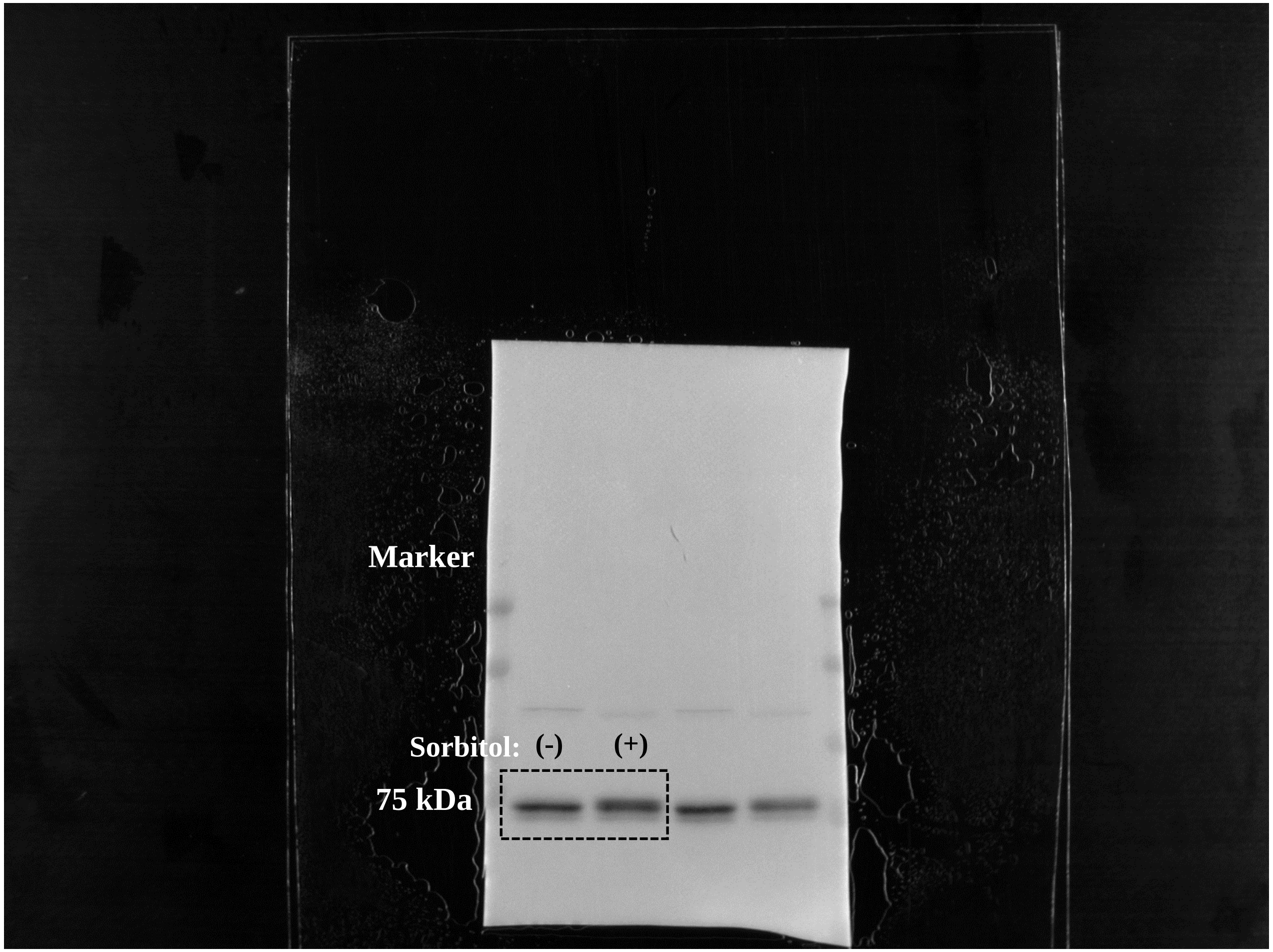

Figure S1D

WB: GAPDH

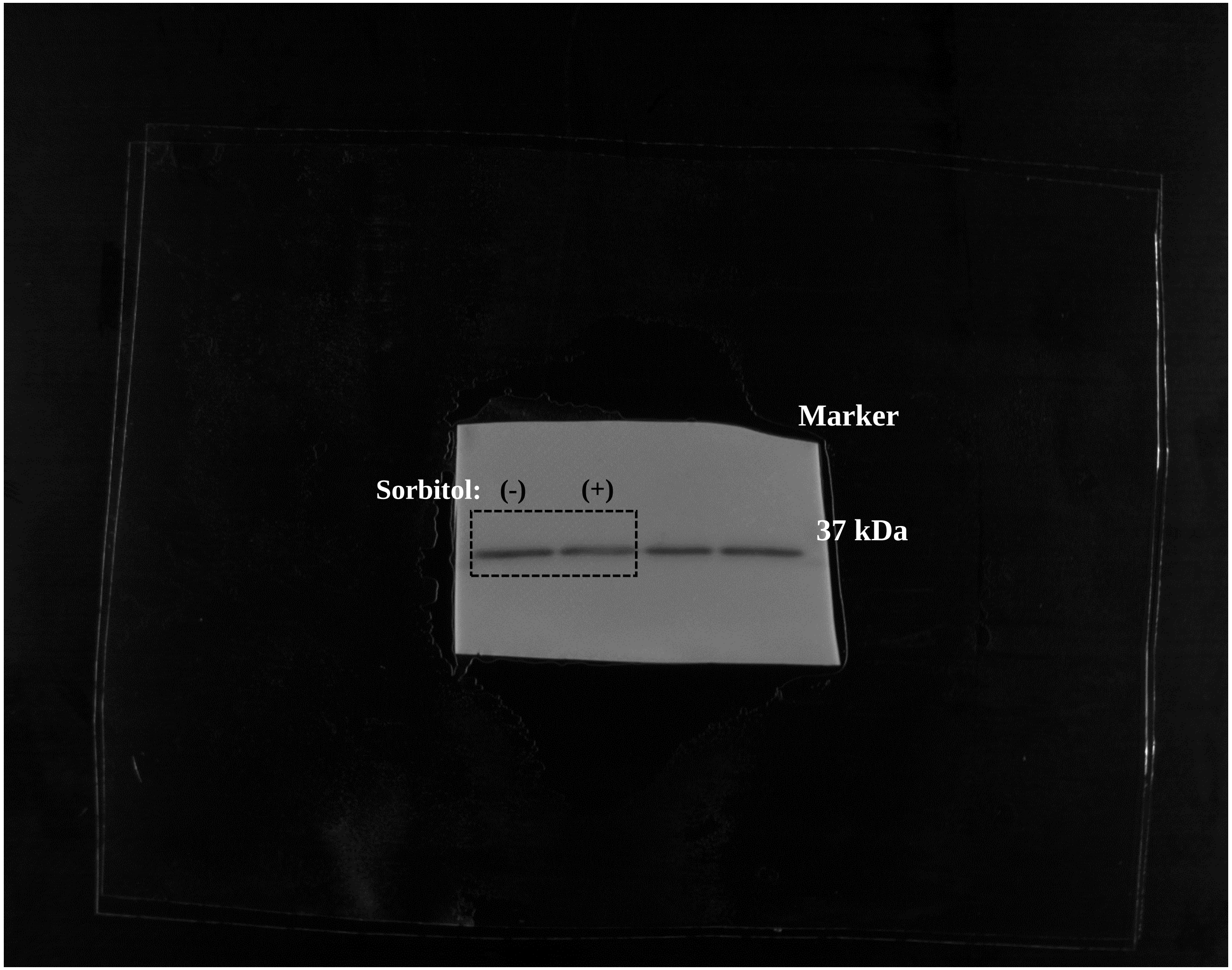

Figure S1E

IP: HA_WB: pS315

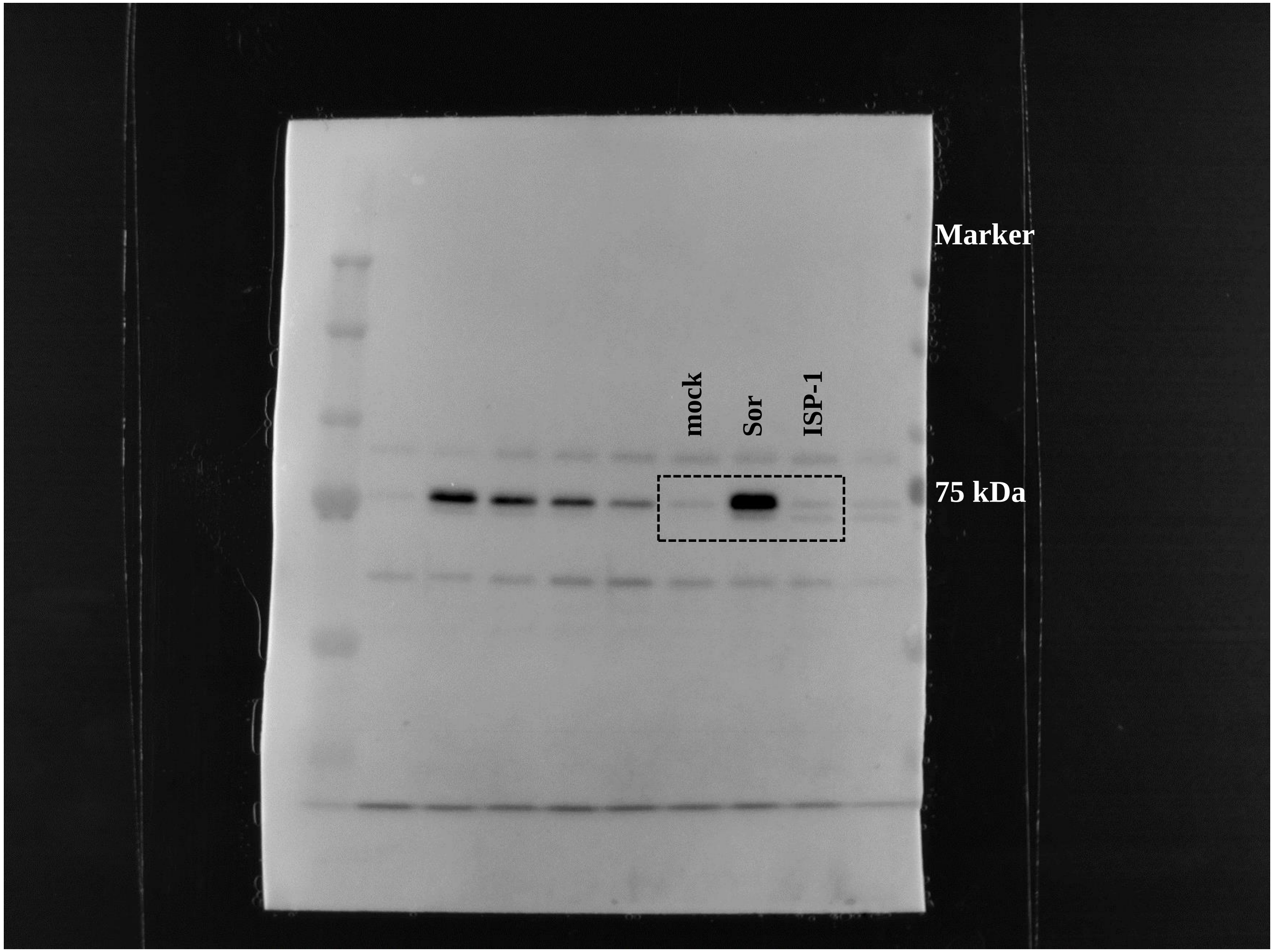

Figure S1E

IP: HA_WB: HA

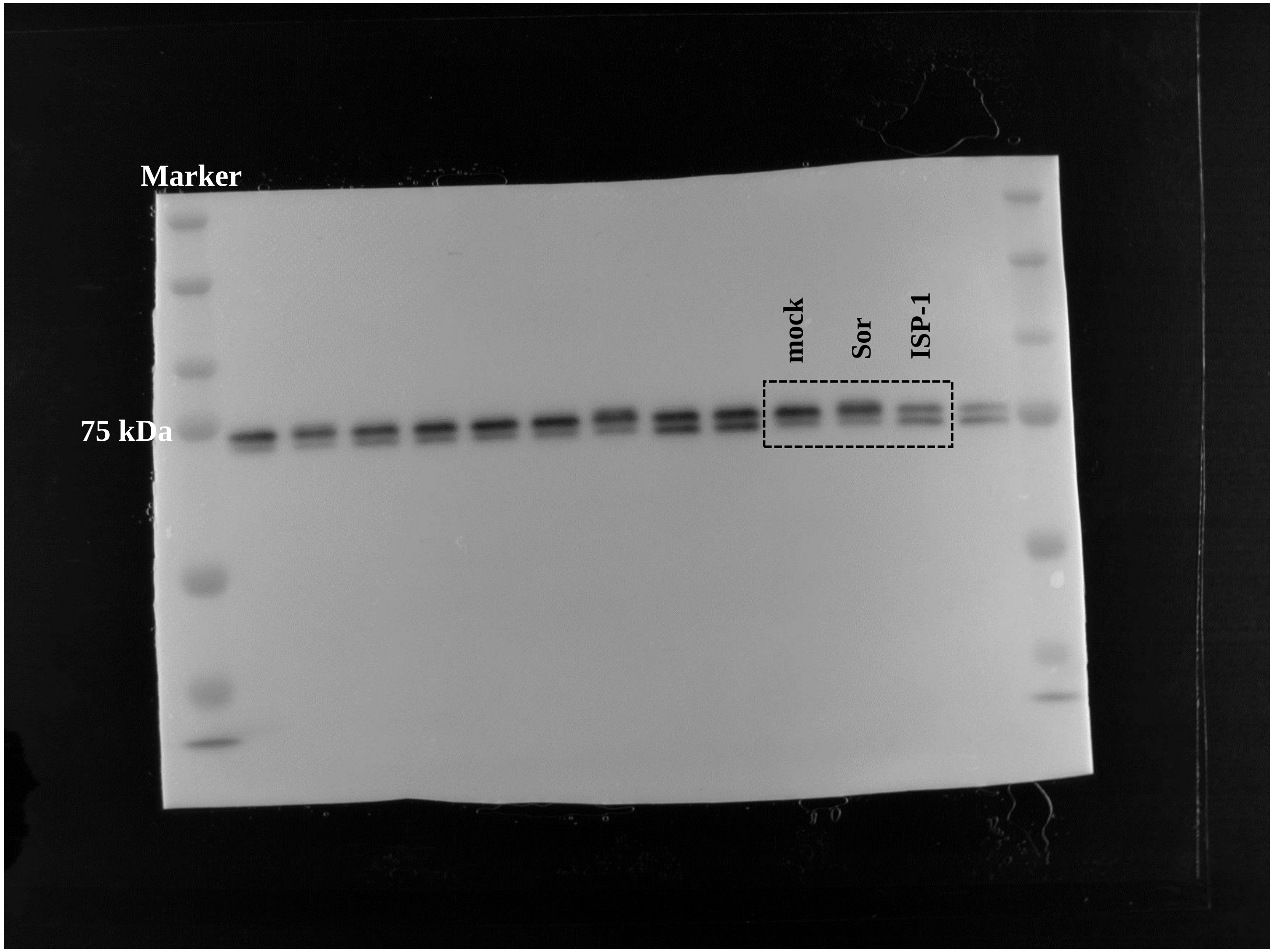

Figure S1E

WB: HA

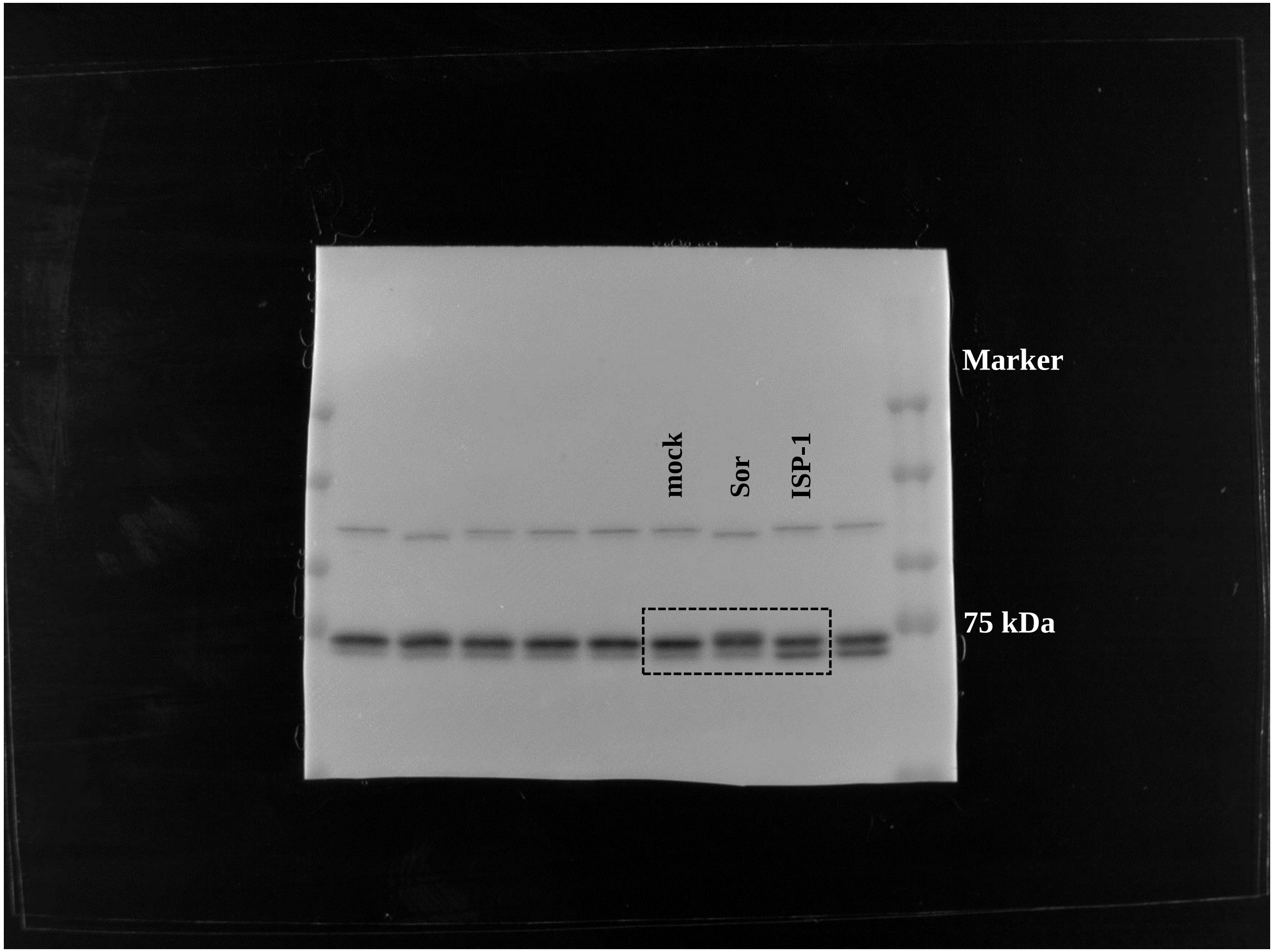

Figure S1E

WB: GAPDH

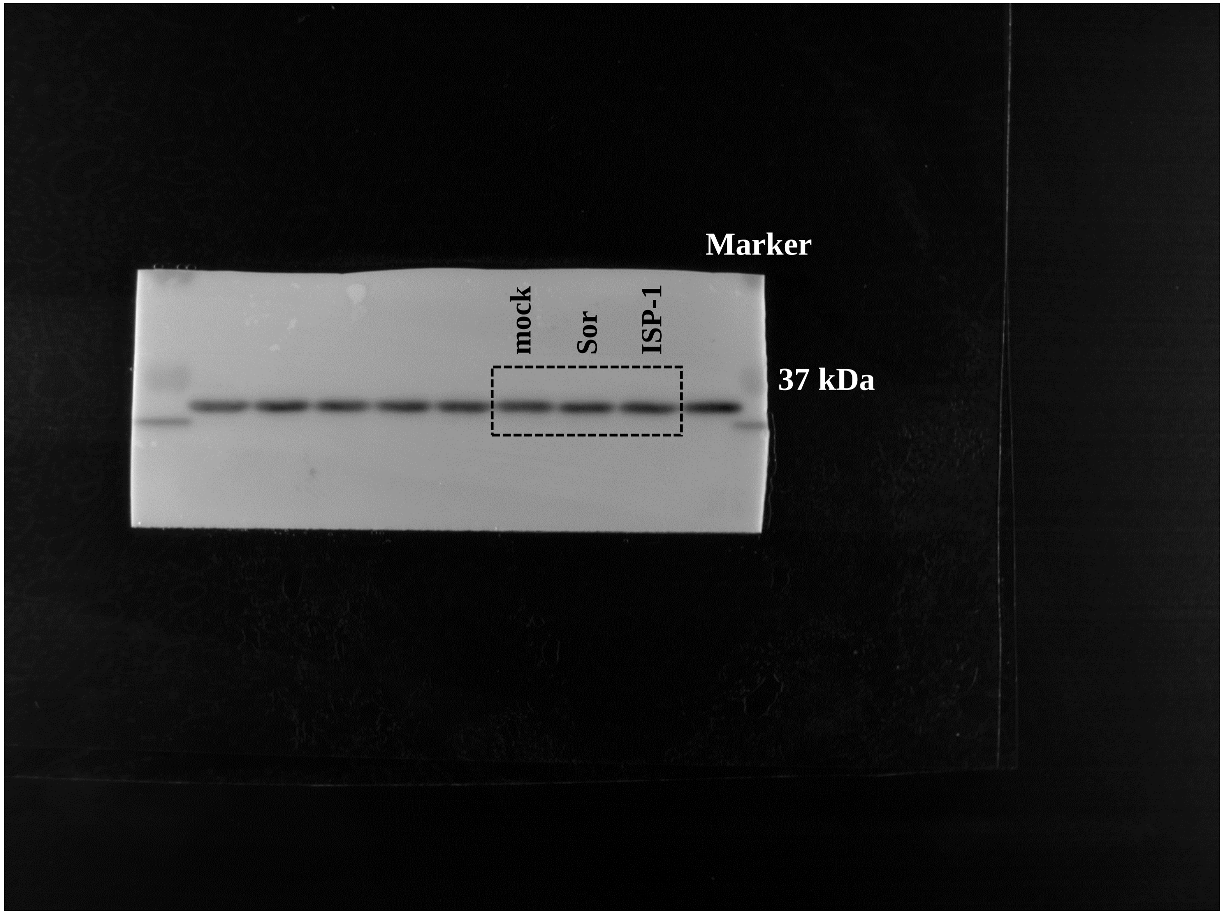

Figure S1F

IP: HA_WB: pS315

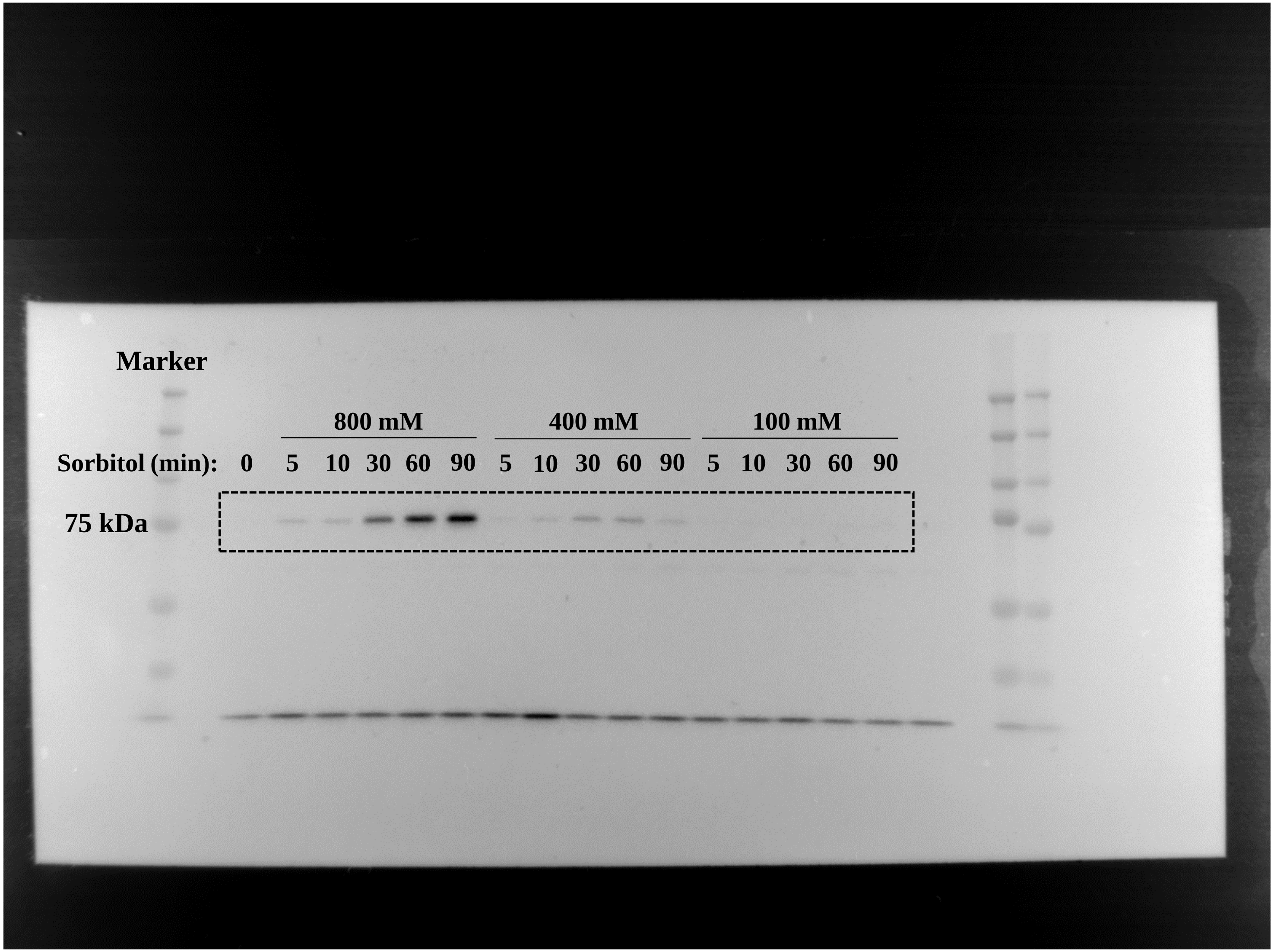

Figure S1F

IP: HA_WB: HA

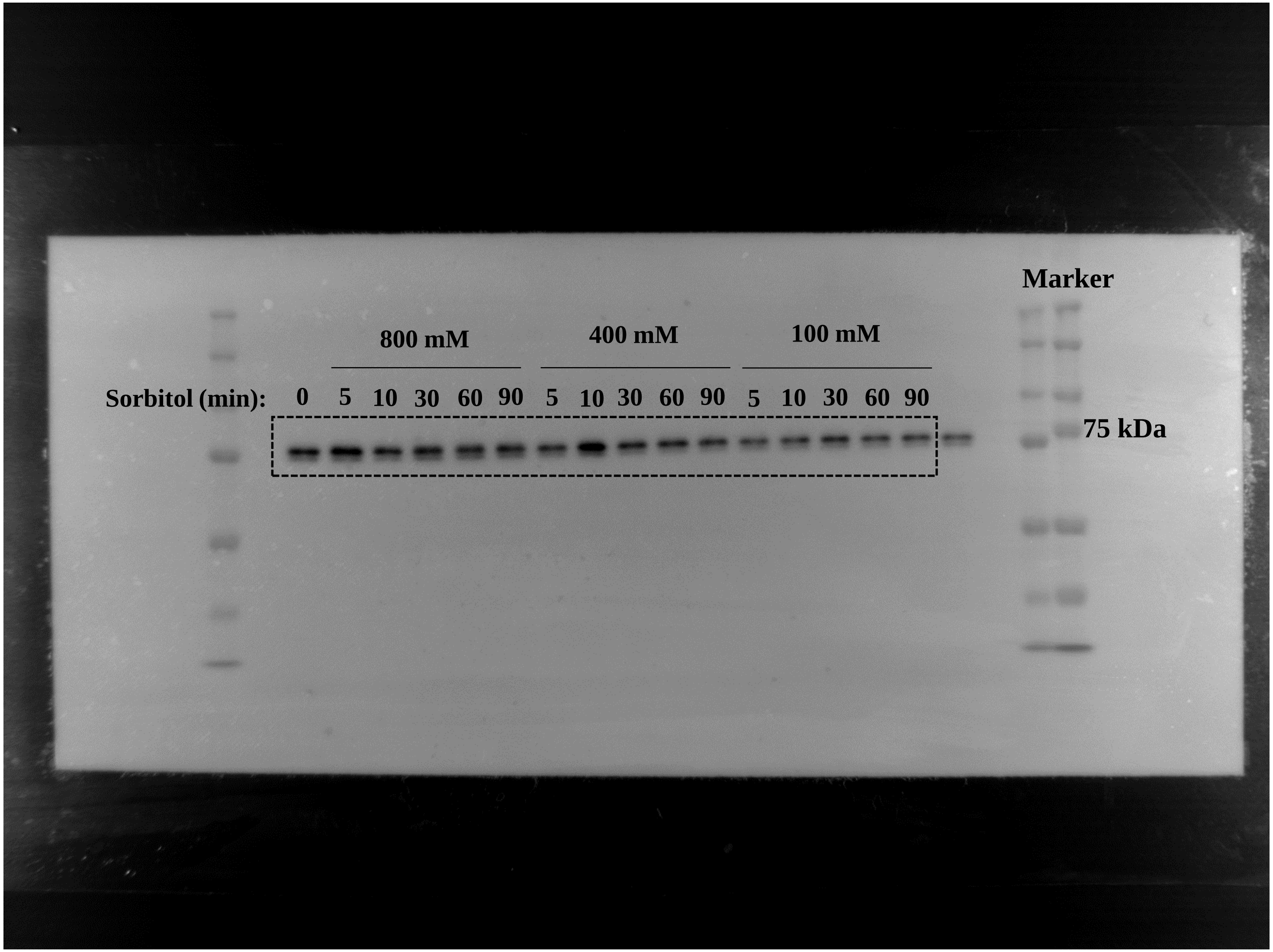

Figure S1F

WB: HA

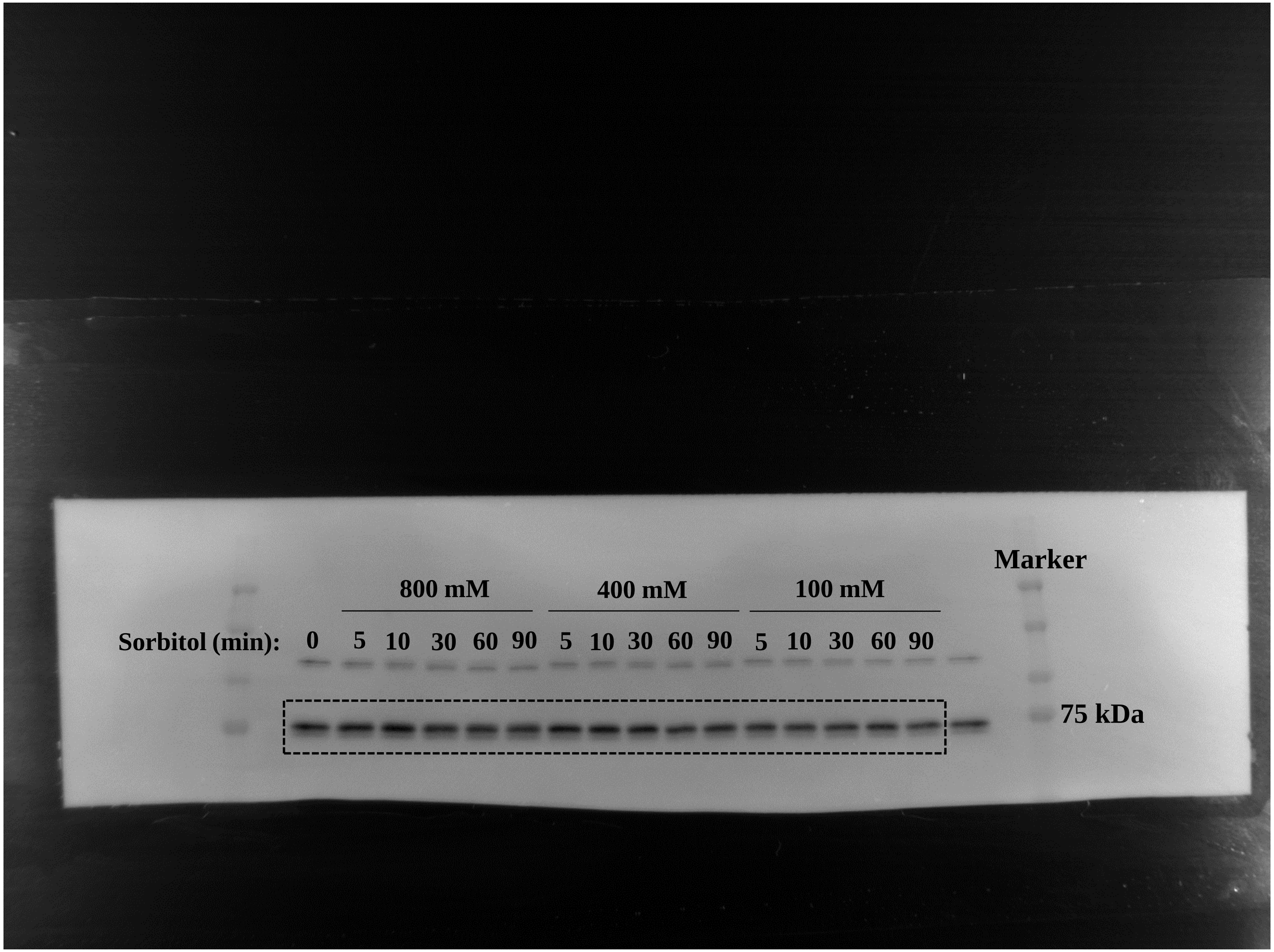

Figure S1F

WB: GAPDH

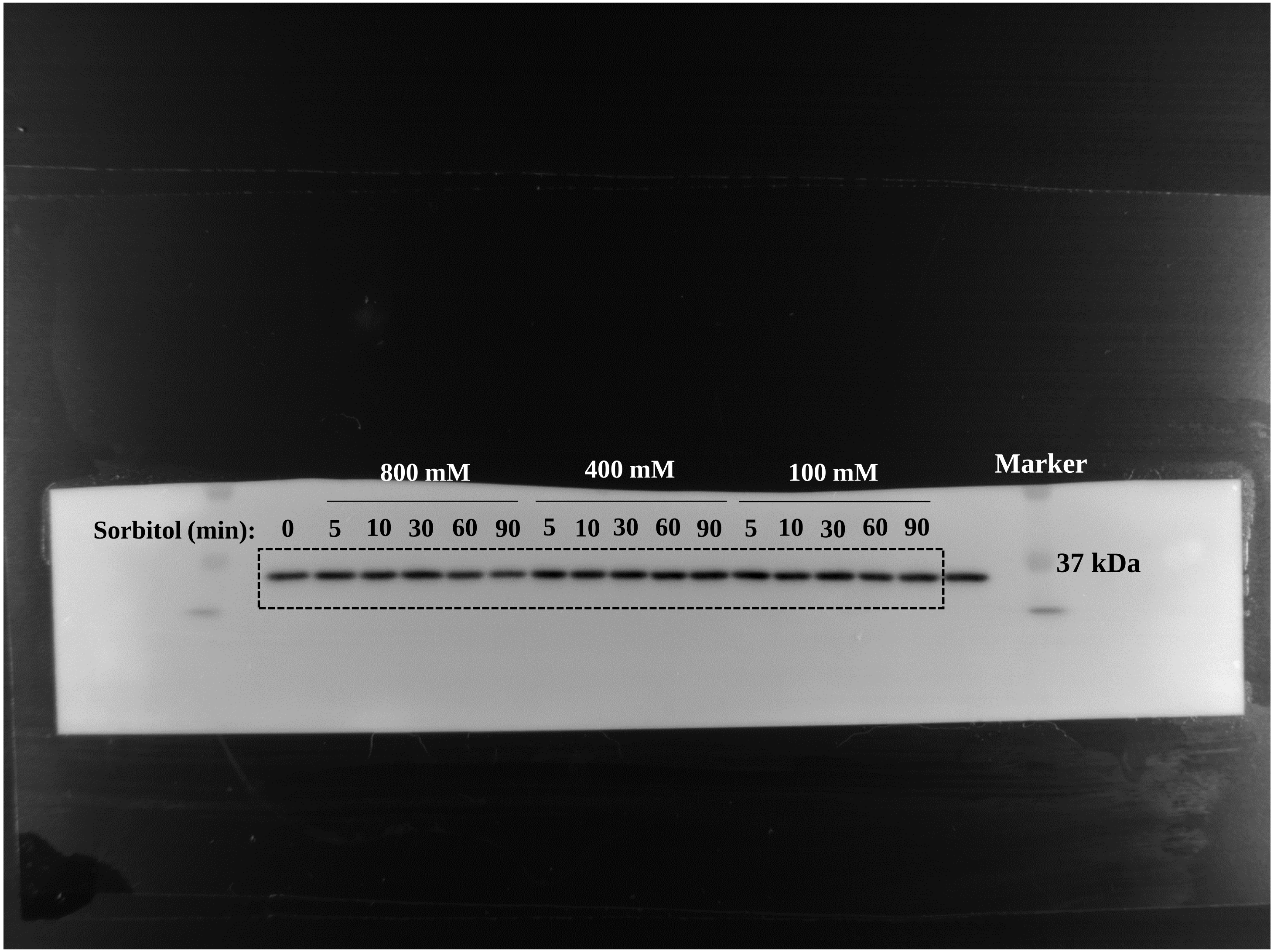

Figure S1G

IP: HA_WB: pS315

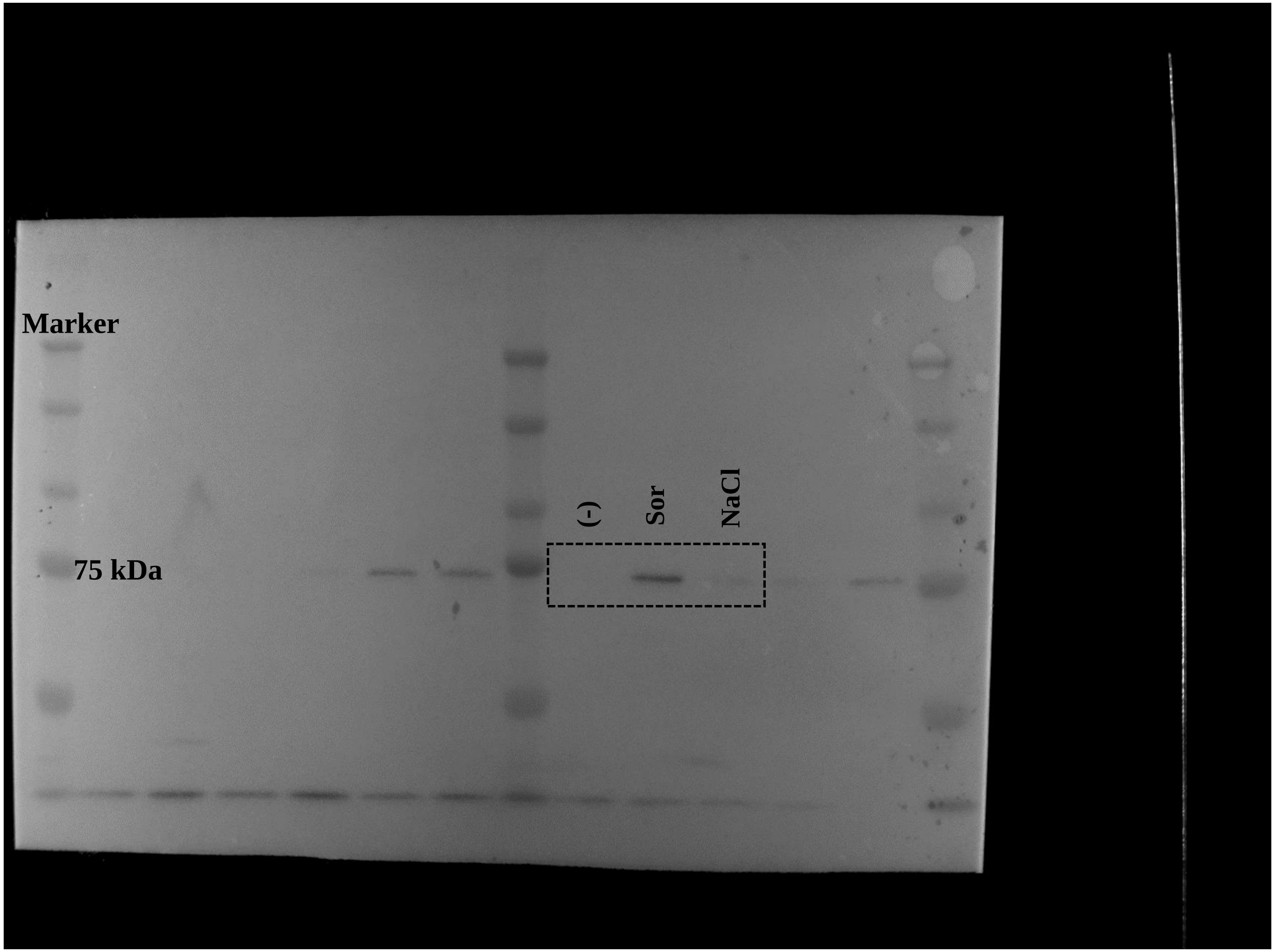

Figure S1G

IP: HA_WB: HA

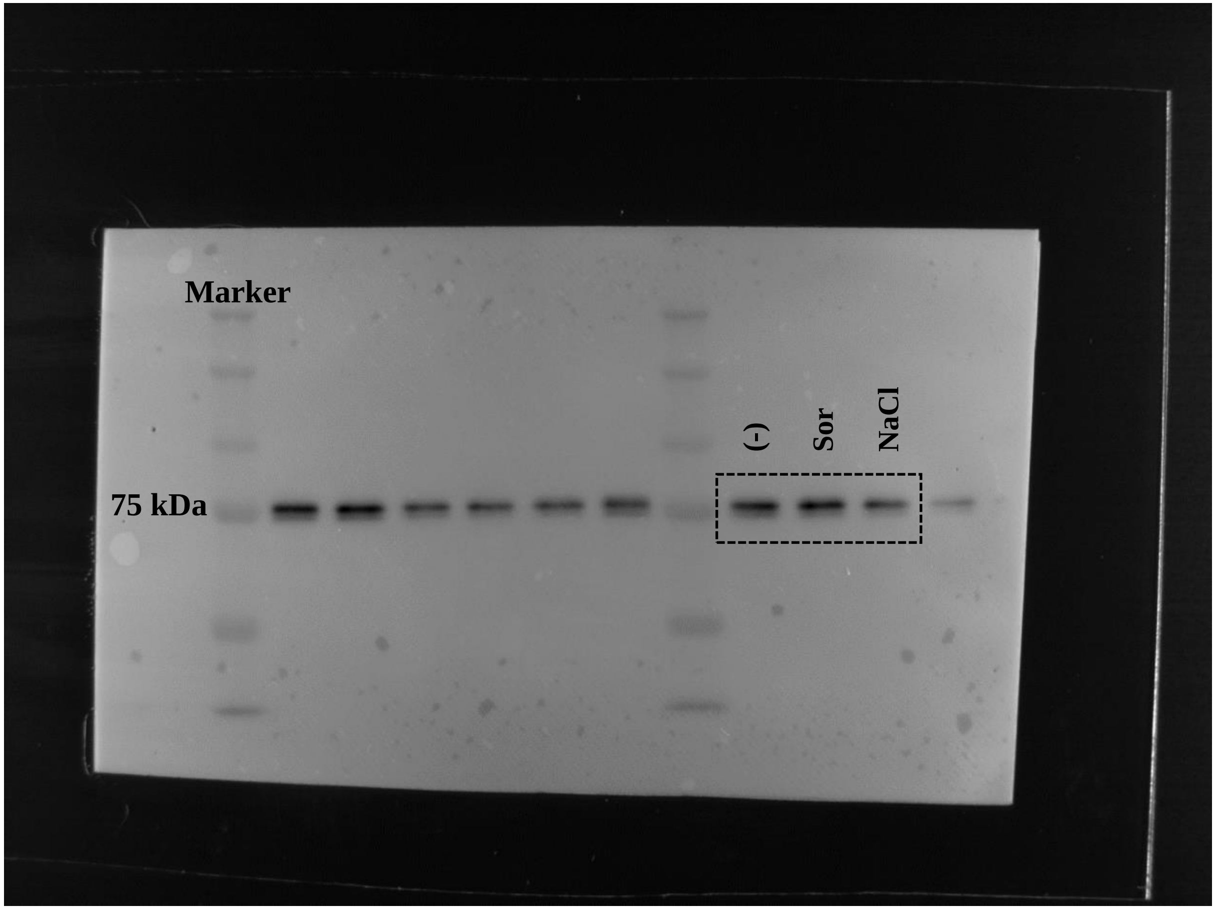

Figure S1G

WB: HA

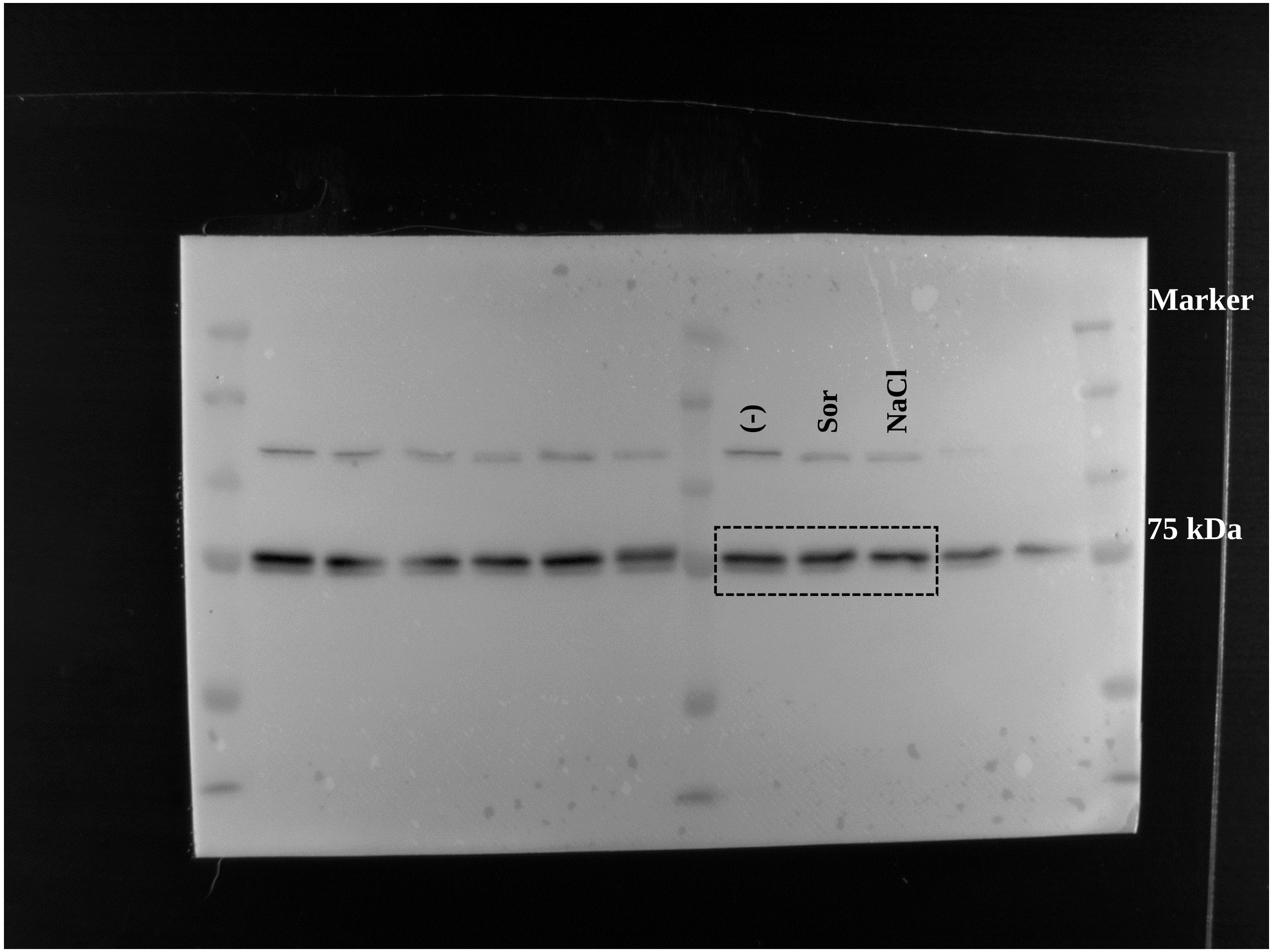

Figure S1G

WB: GAPDH

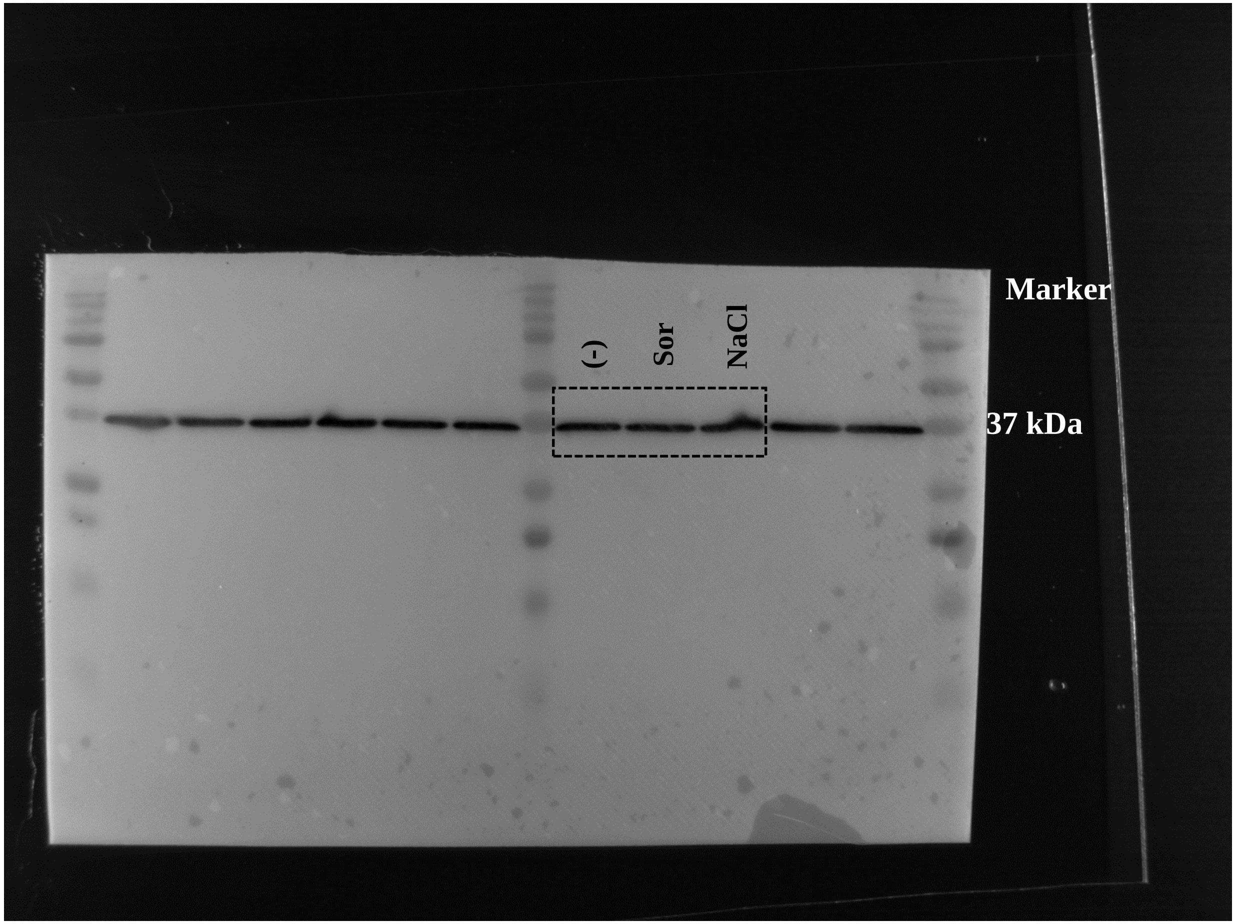

Figure S2A

IP: HA_WB: pS315

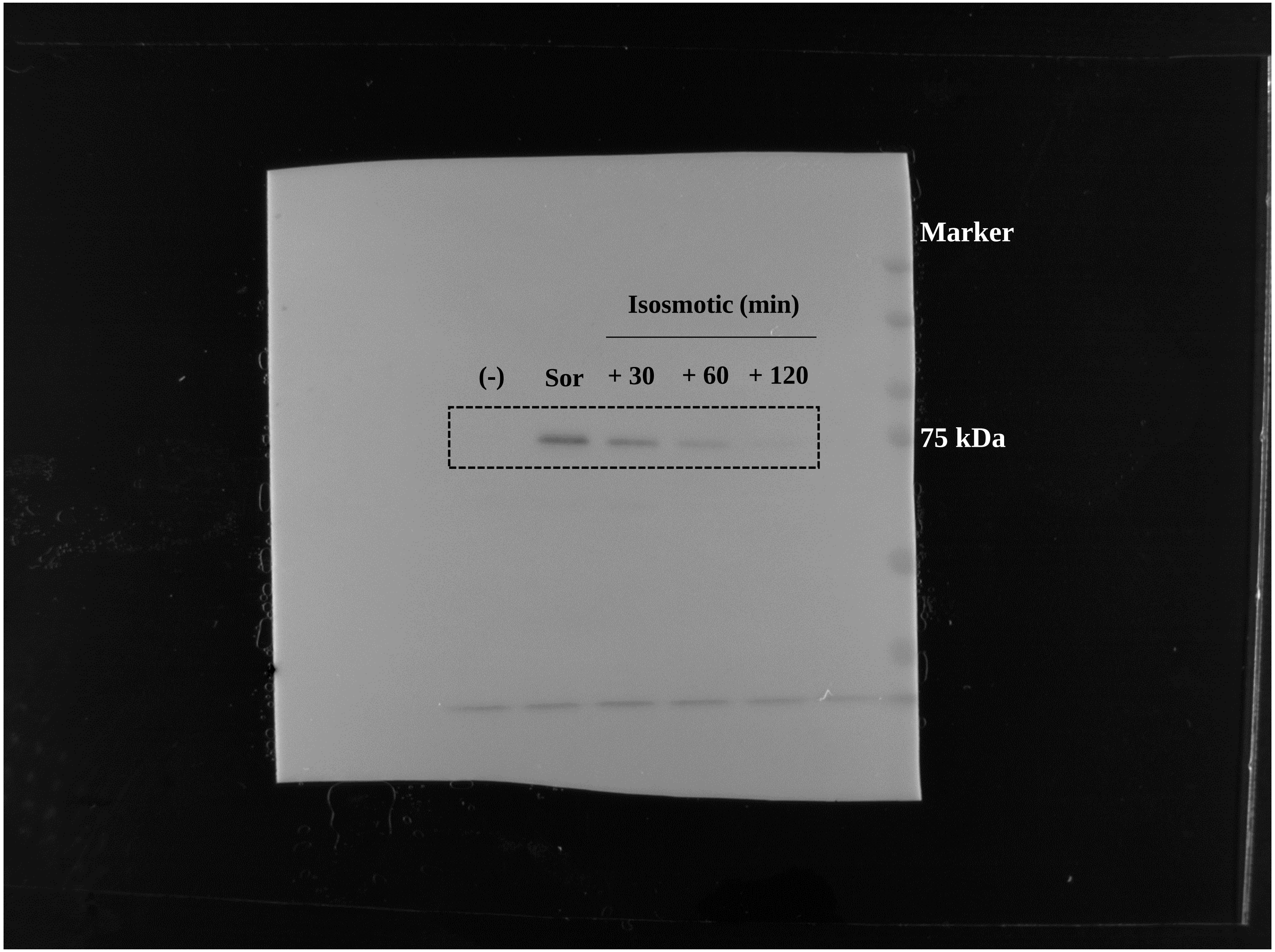

Figure S2A

IP: HA_WB: HA

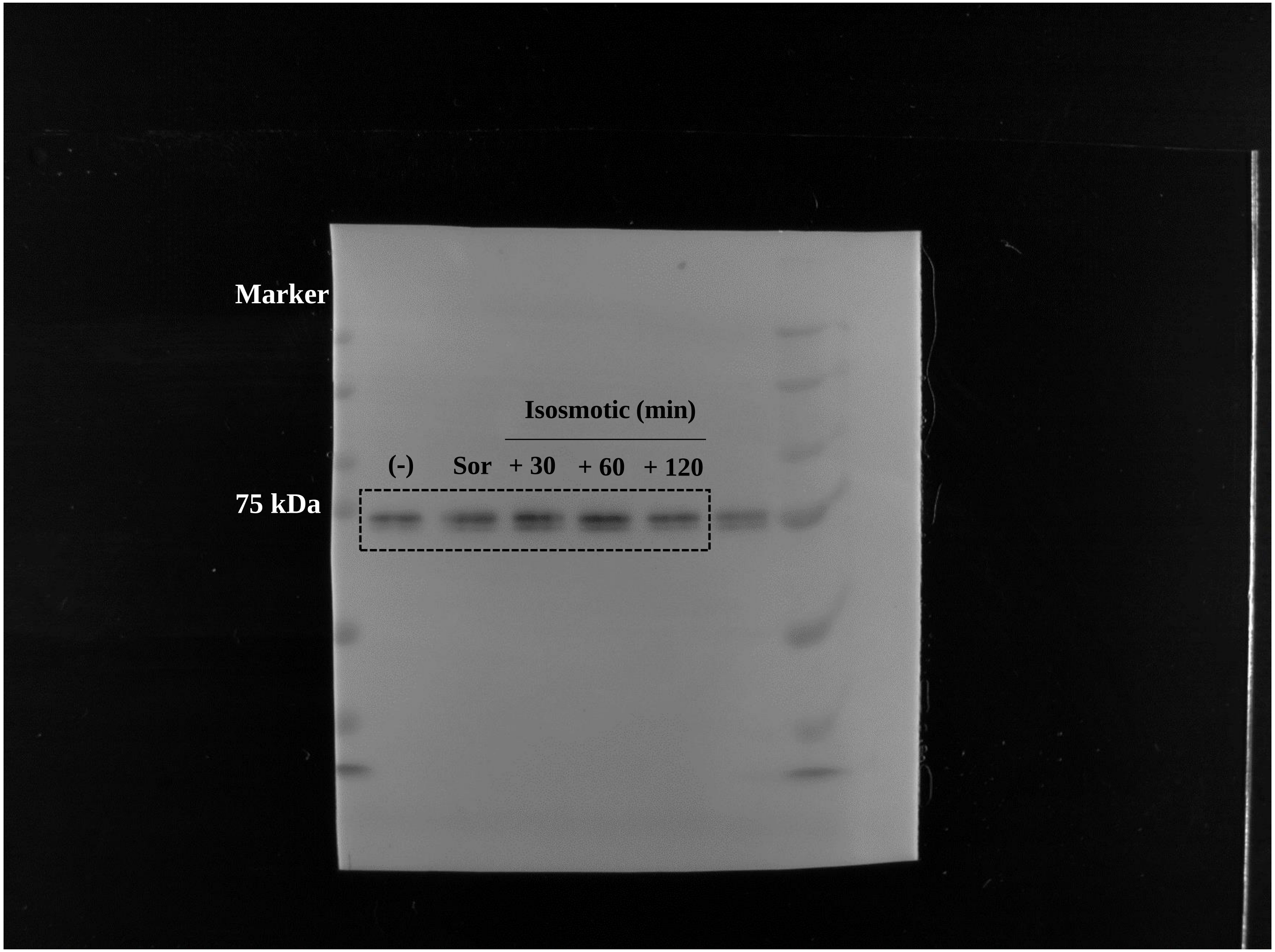

Figure S2A

WB: HA

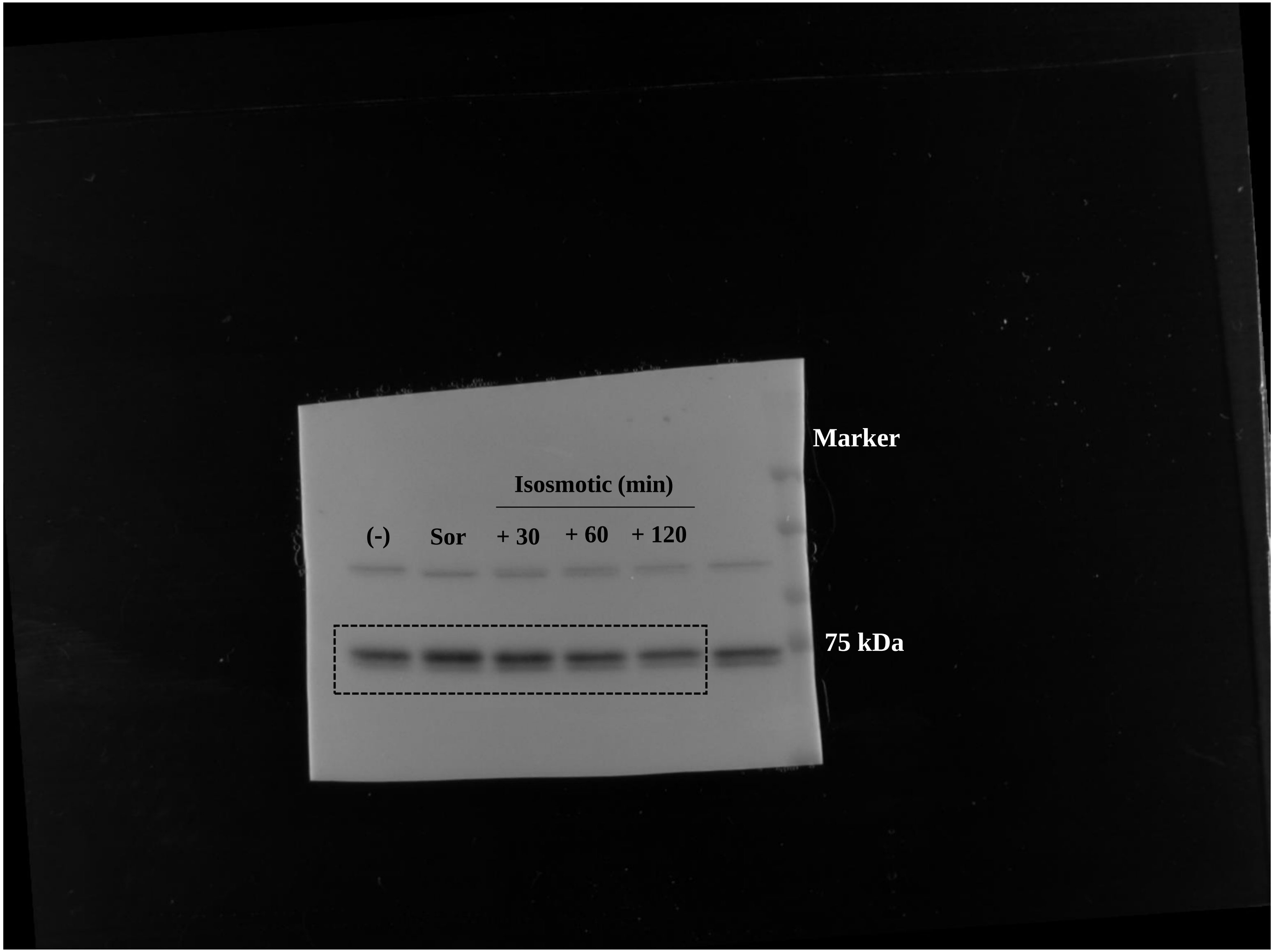

Figure S2A

WB: GAPDH

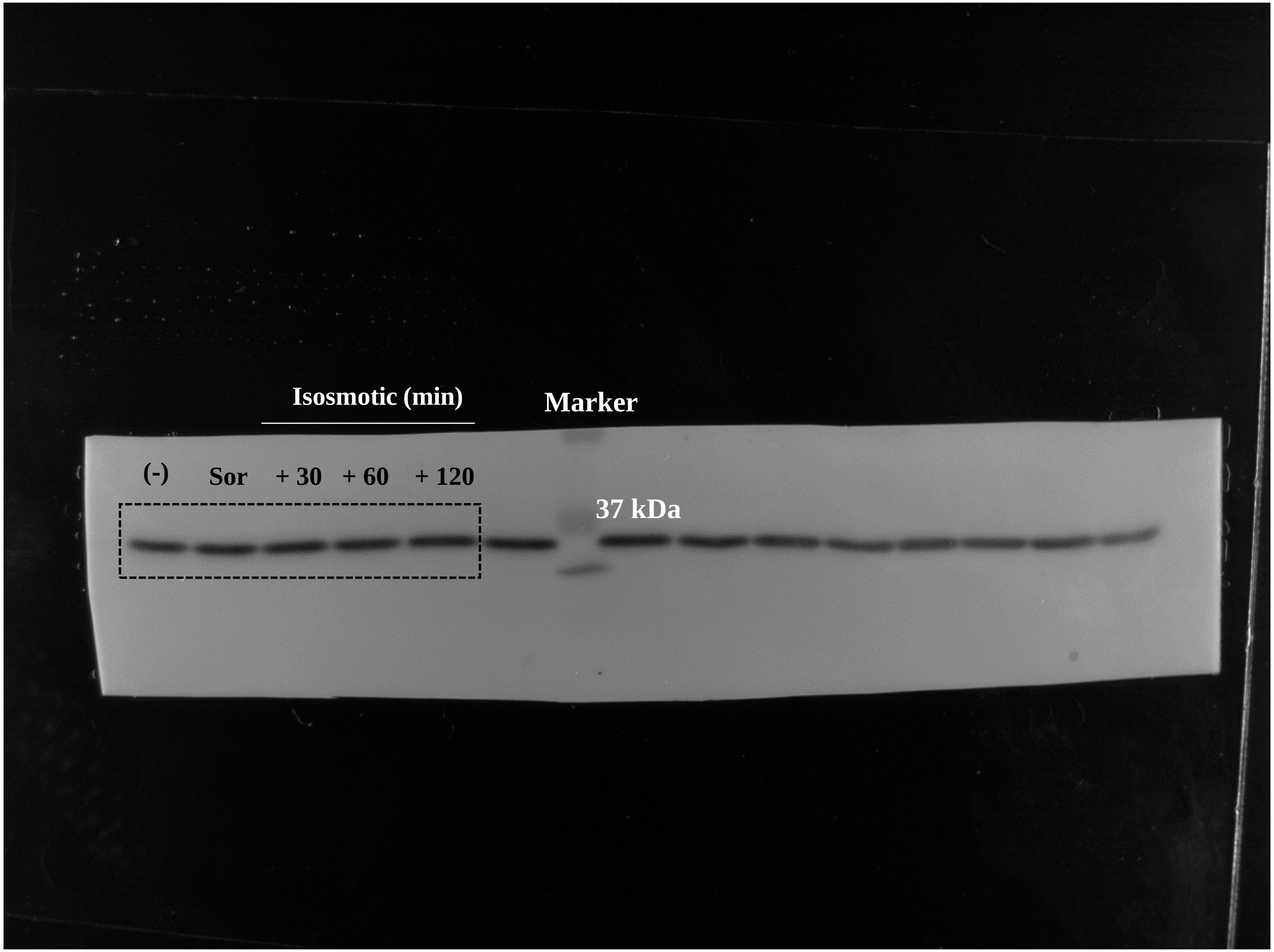

Figure S2B

IP: HA_WB: pS315

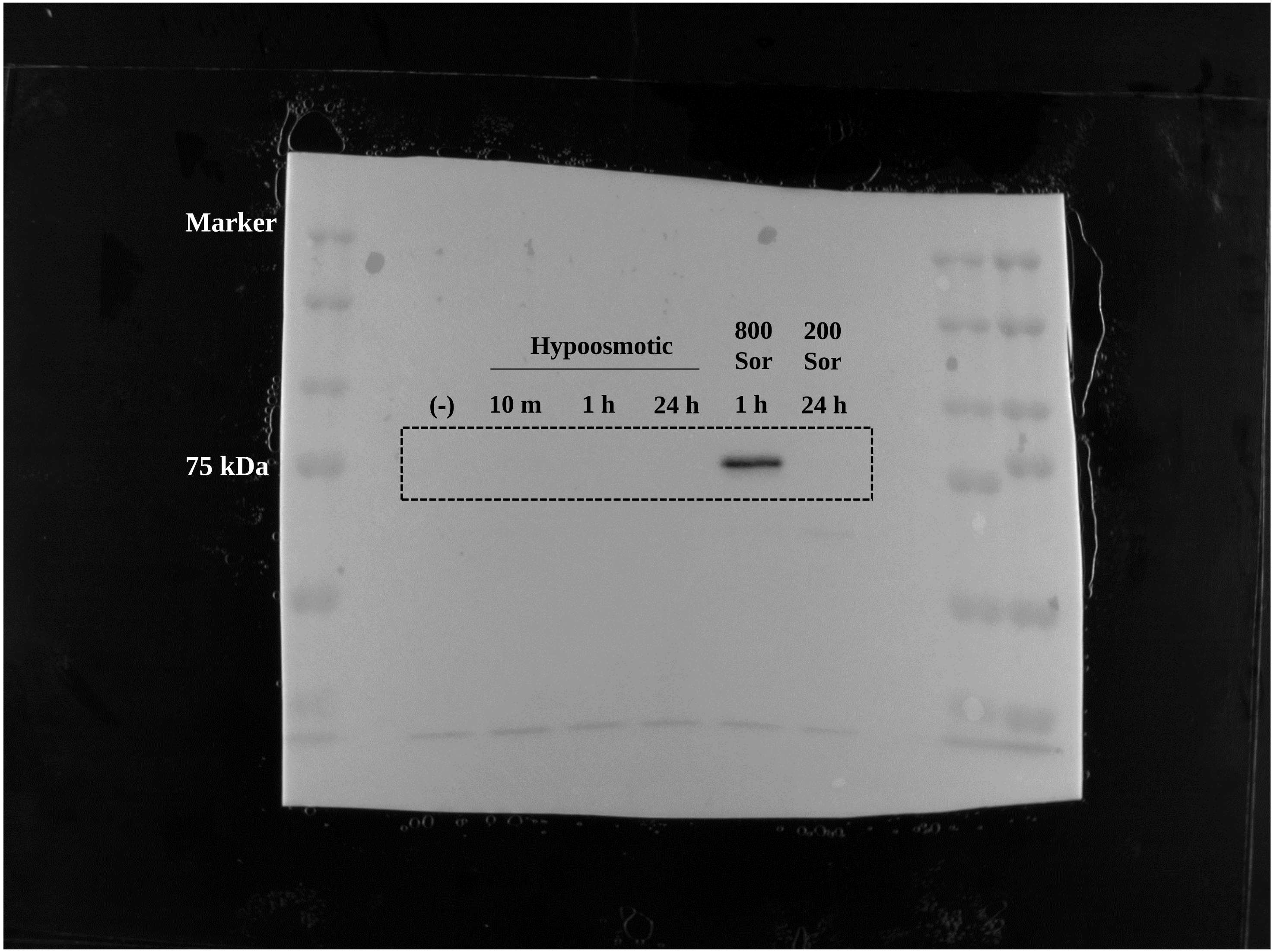

Figure S2B

IP: HA_WB: HA

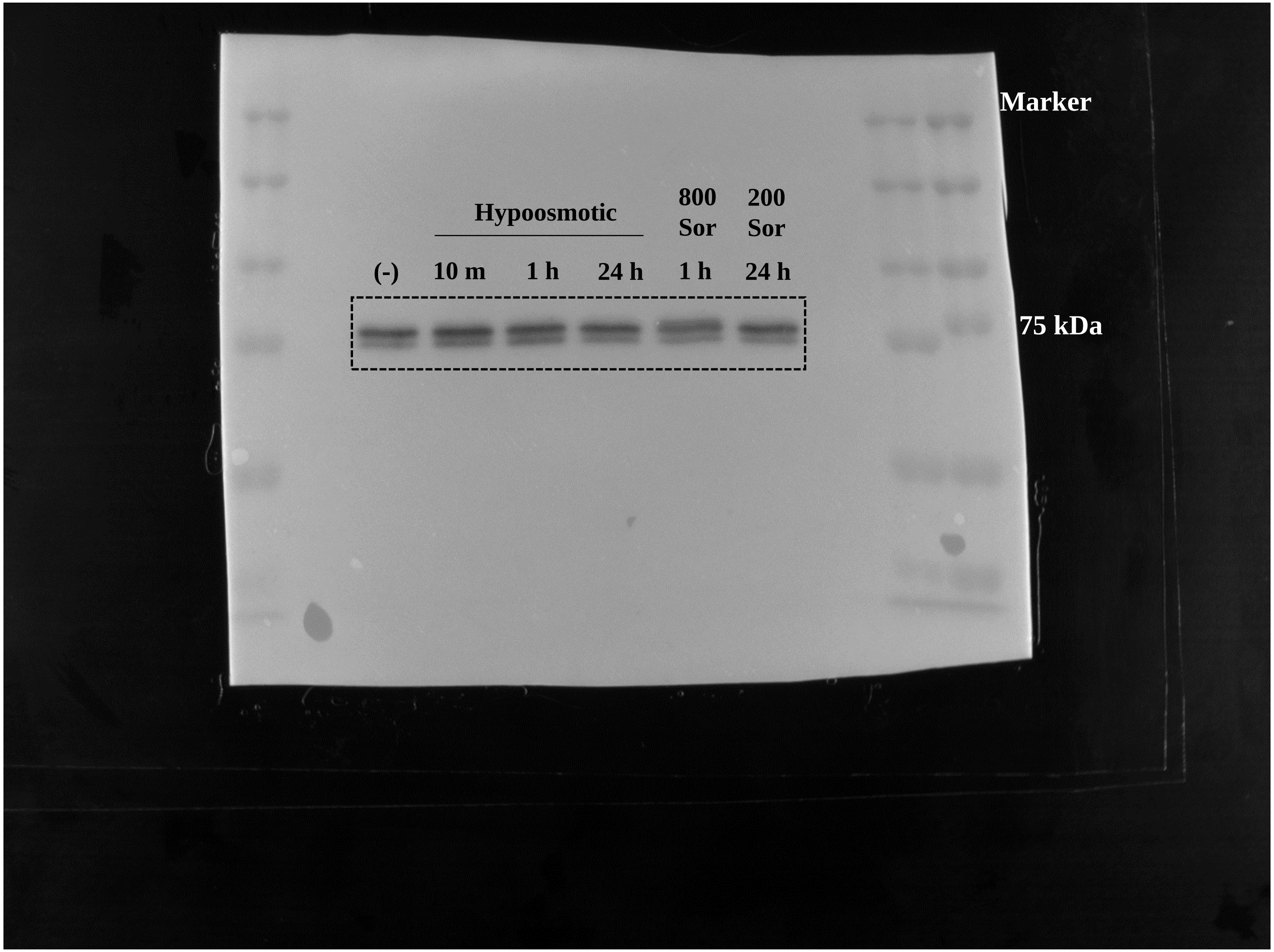

Figure S2B

WB: HA

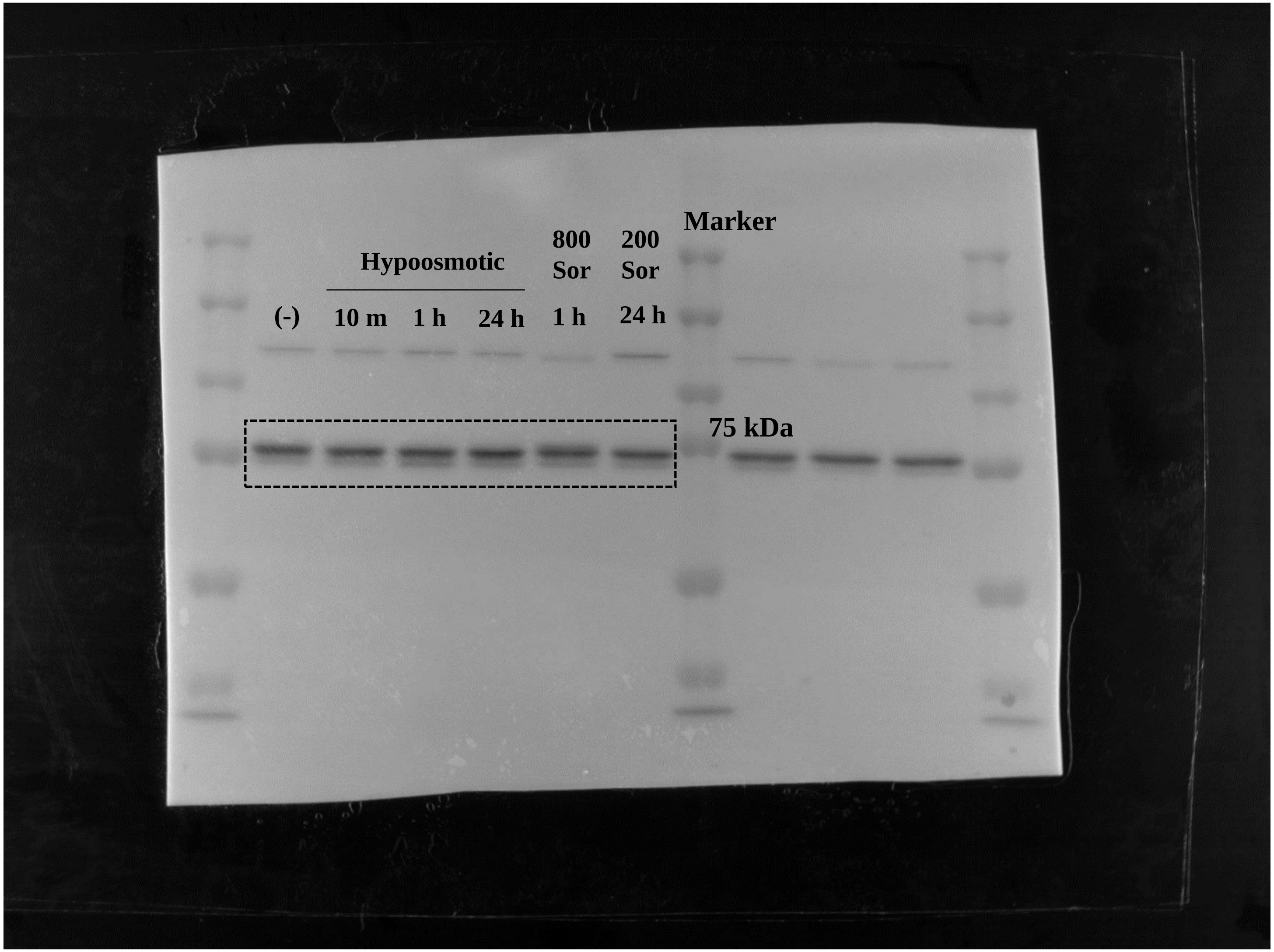

Figure S2B

WB: GAPDH

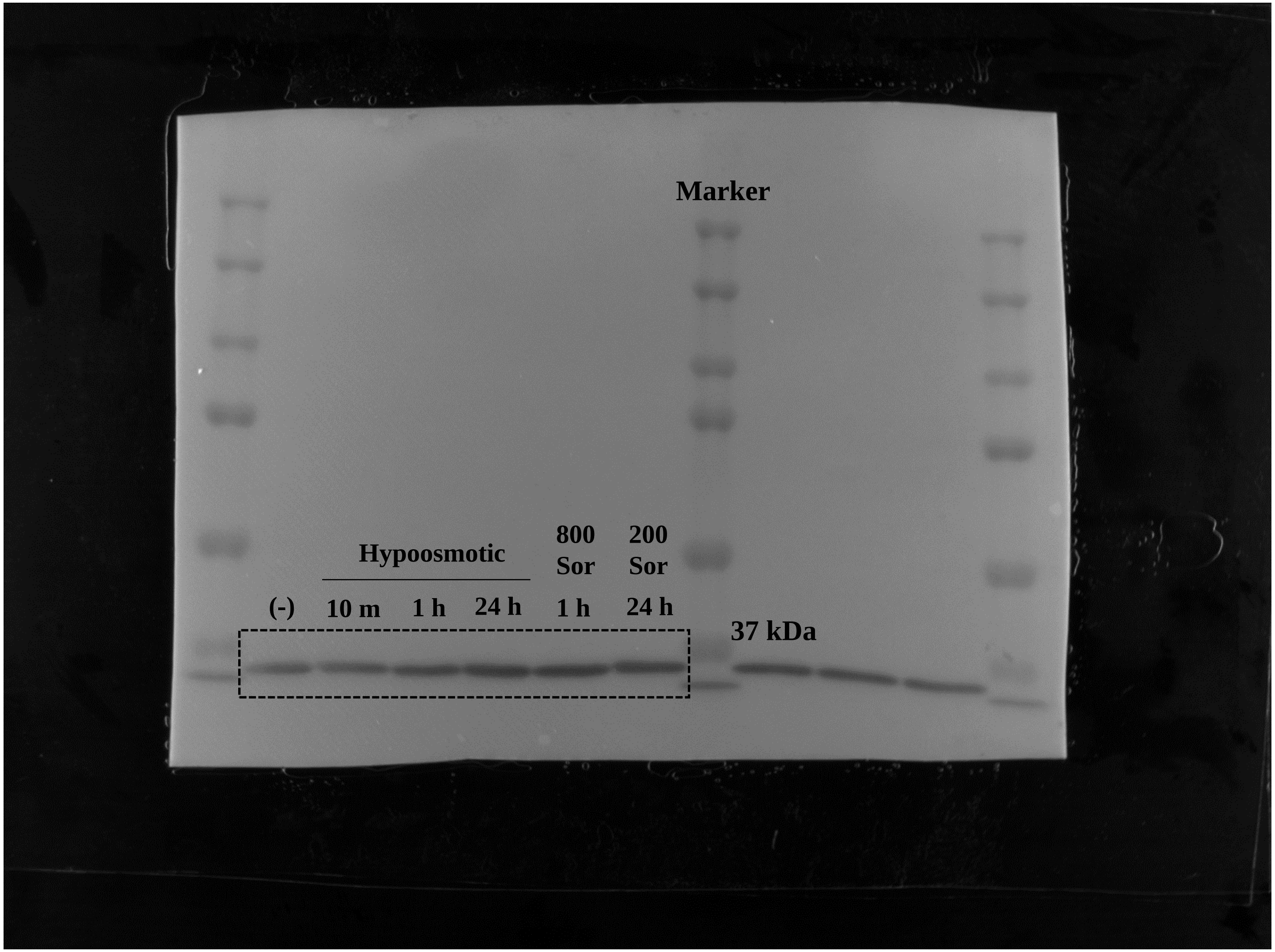

Figure S3A

WB: CERT

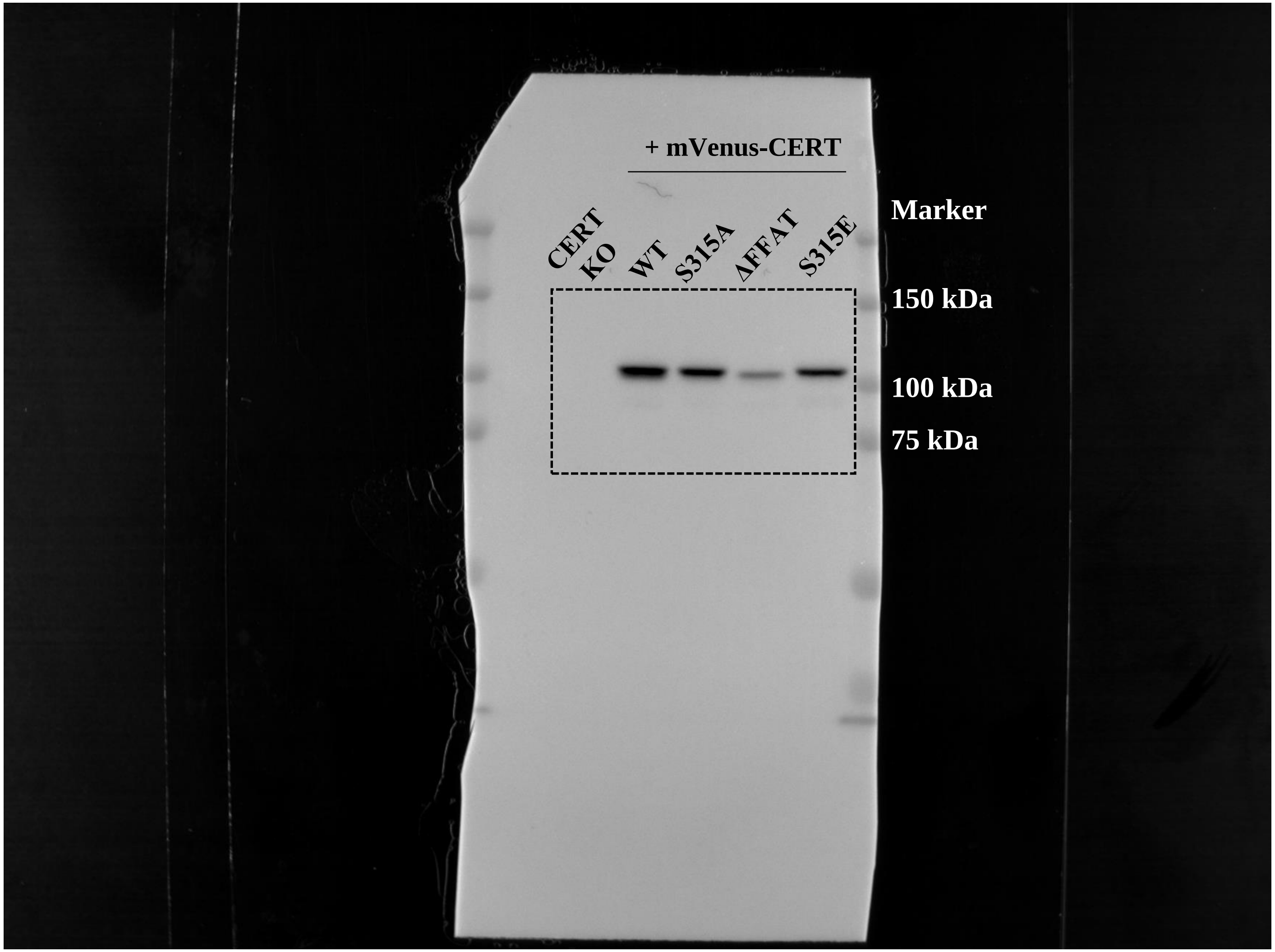

Figure S3A

WB: GFP

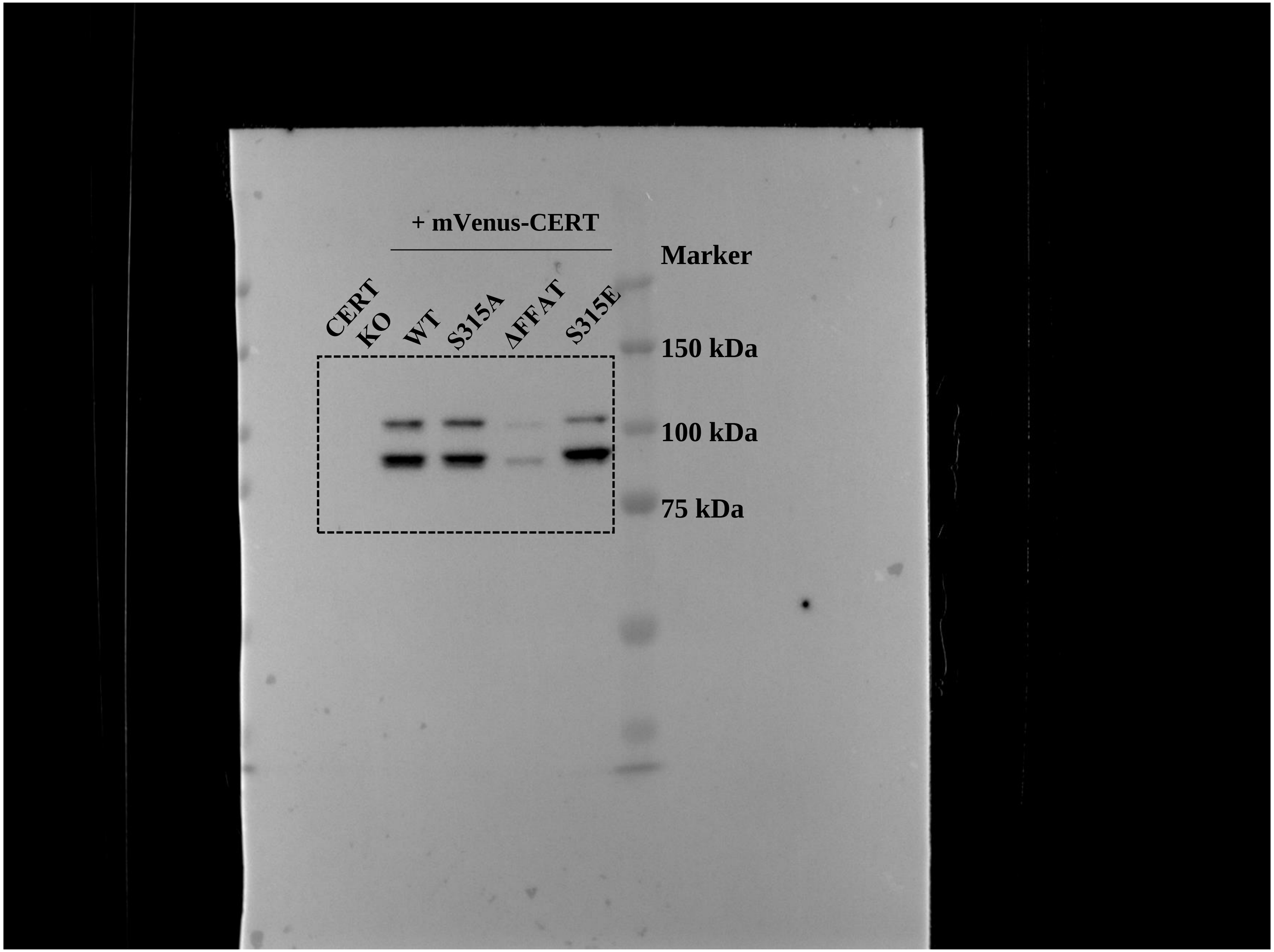

Figure S3A

WB: CERT(c-terminus epitope)

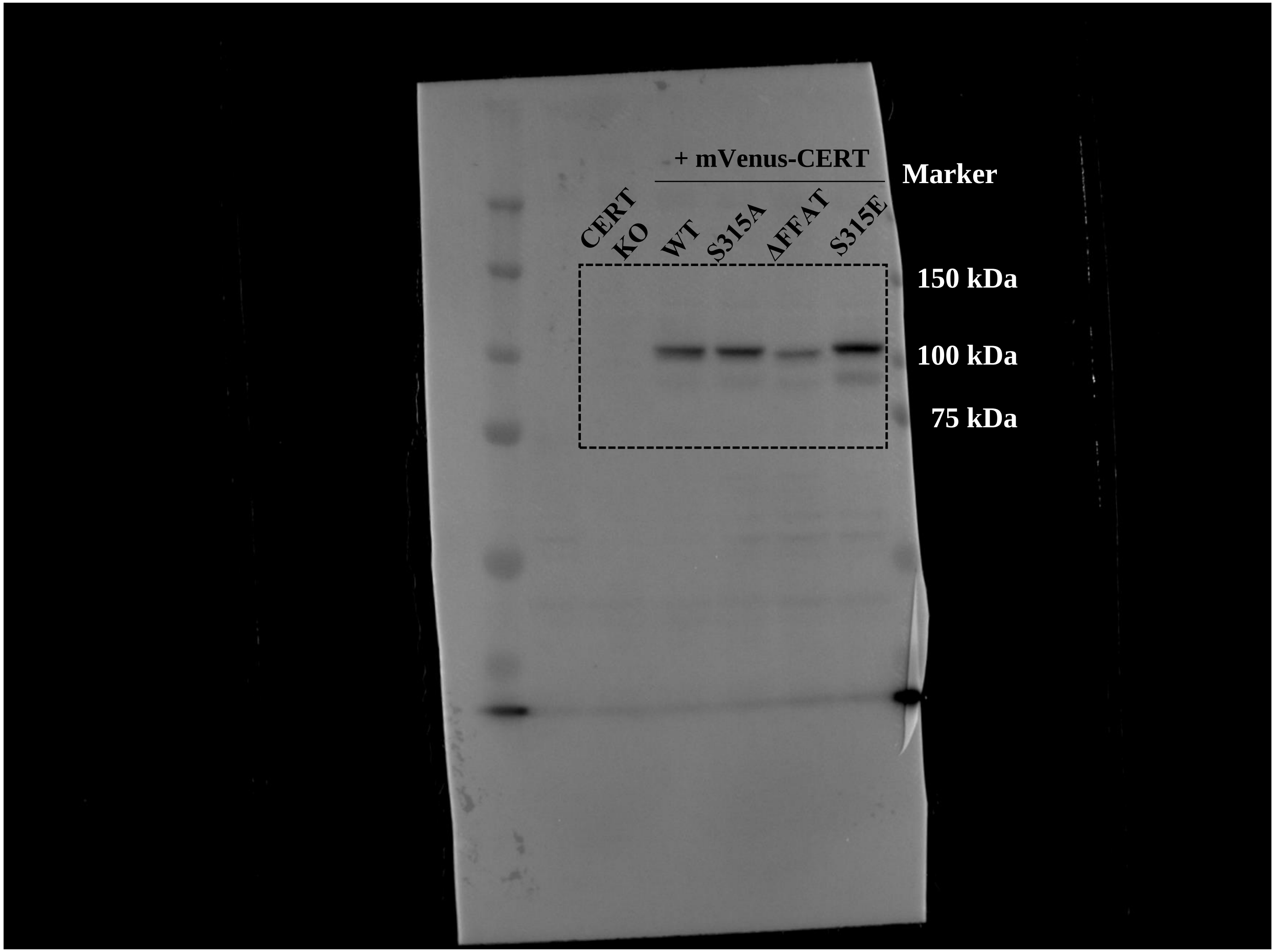

Figure S3A

WB: GAPDH

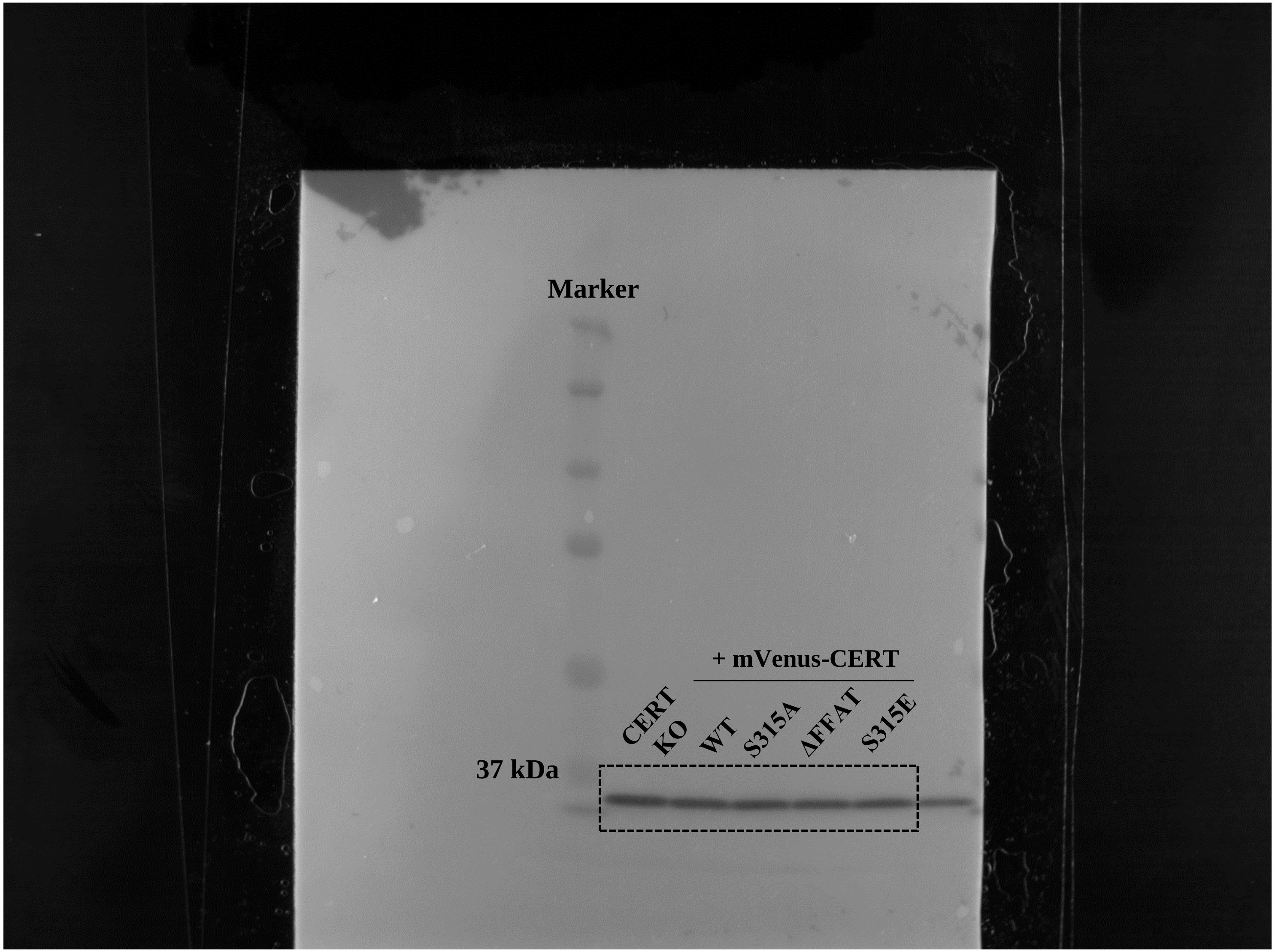

Figure S3B

IP: GFP_WB: pS315

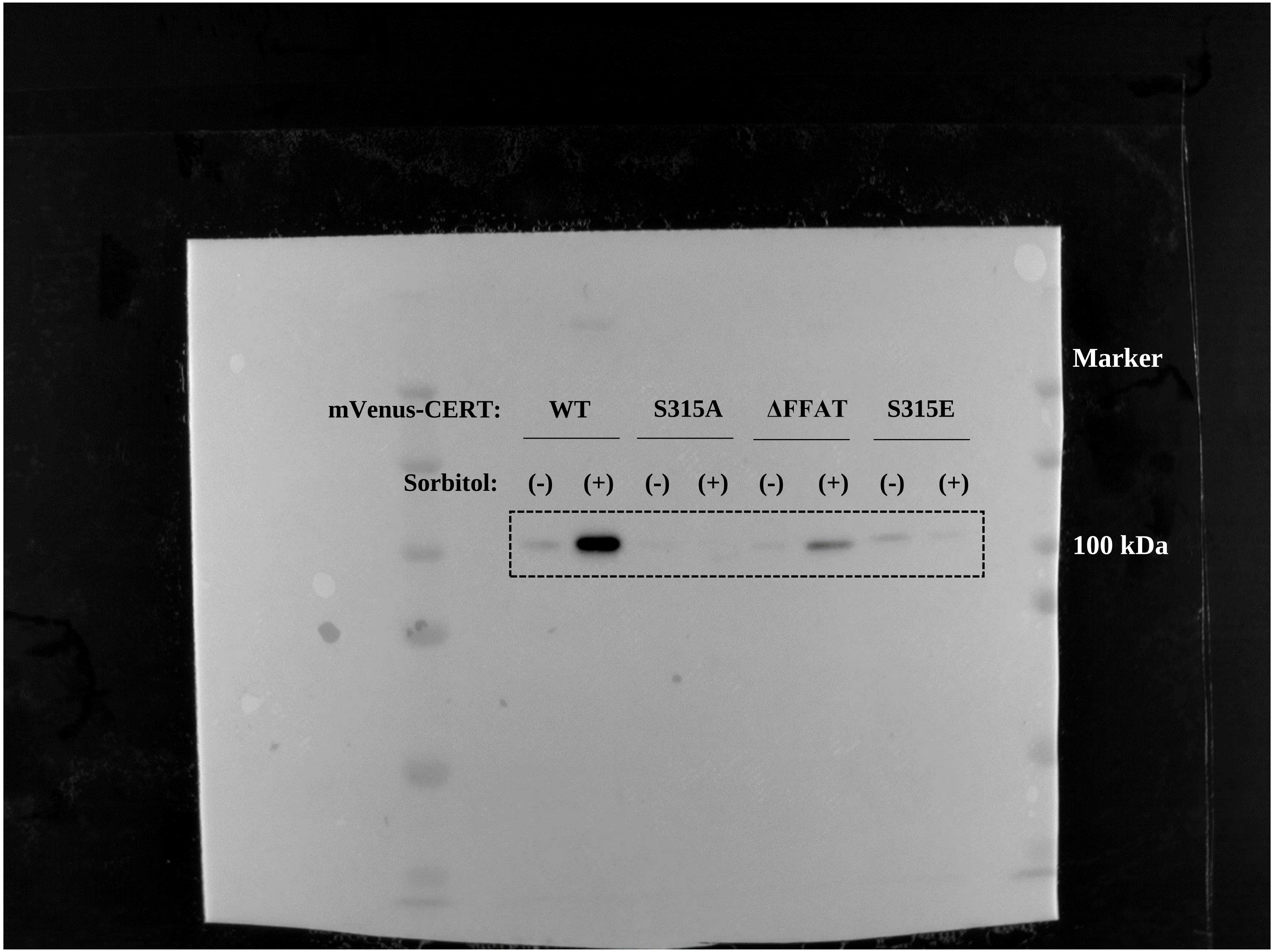

Figure S3B

IP: GFP_WB: VAP-A

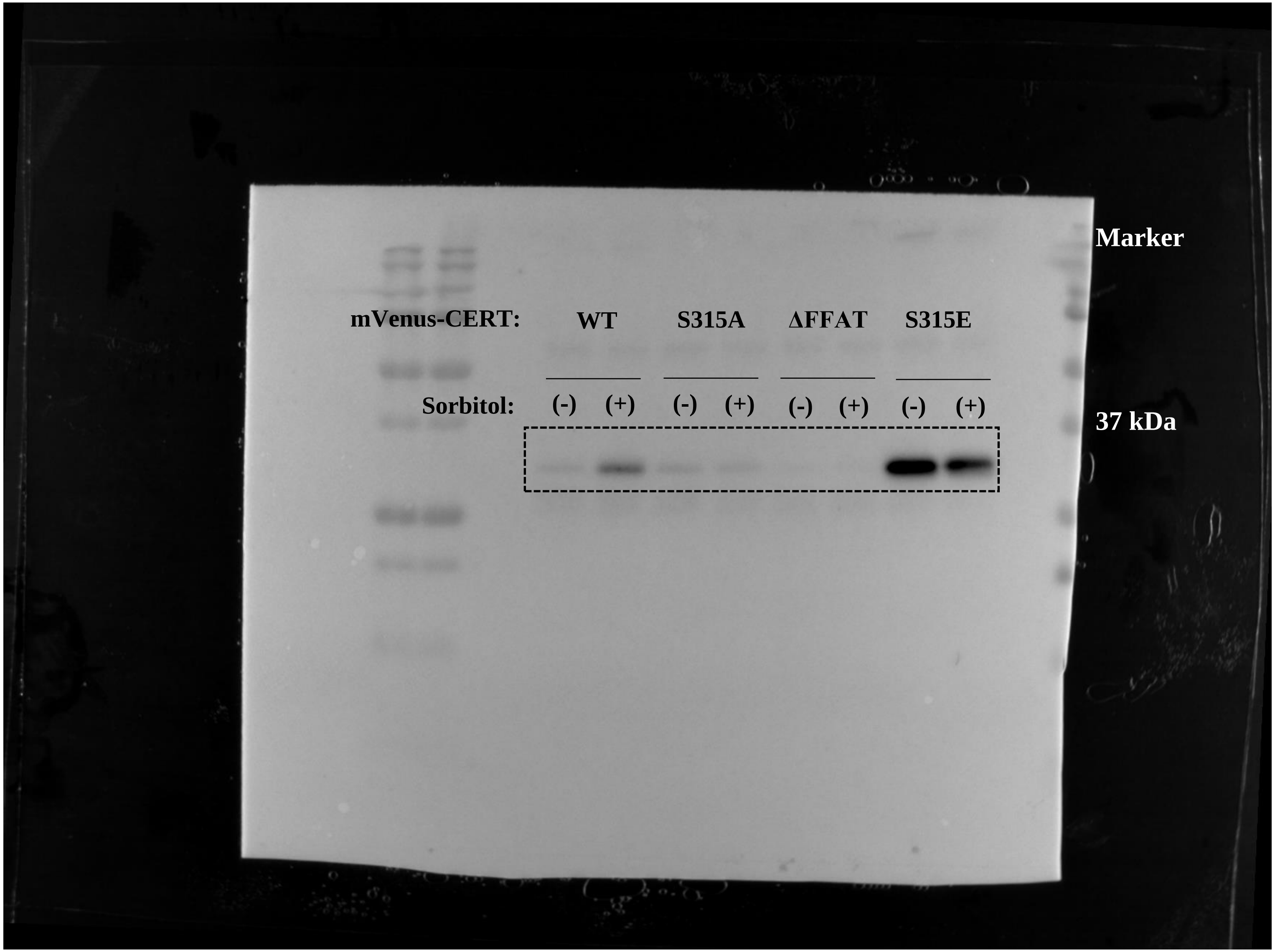

Figure S3B

IP: GFP_WB: CERT

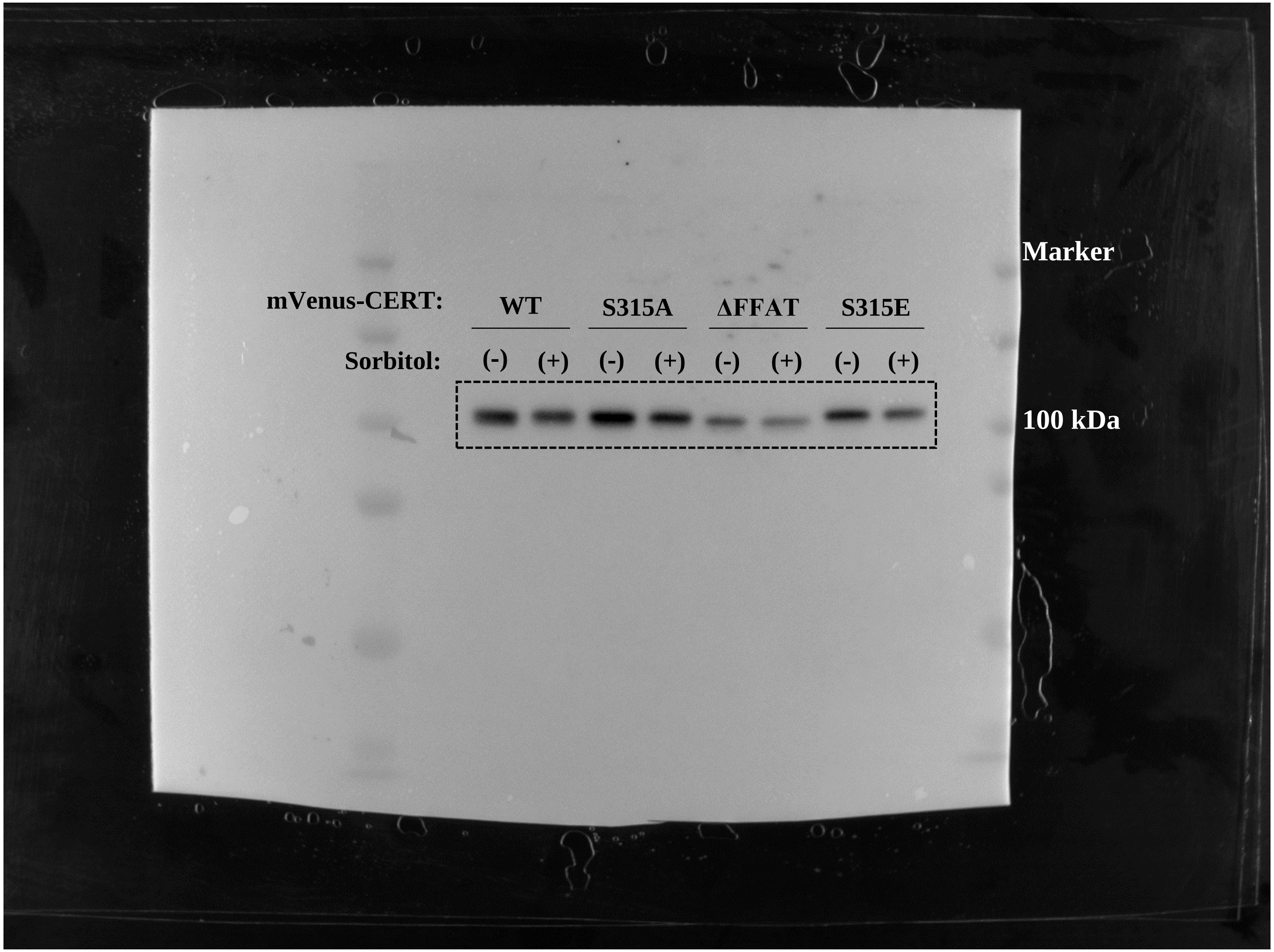

Figure S3B

WB: CERT

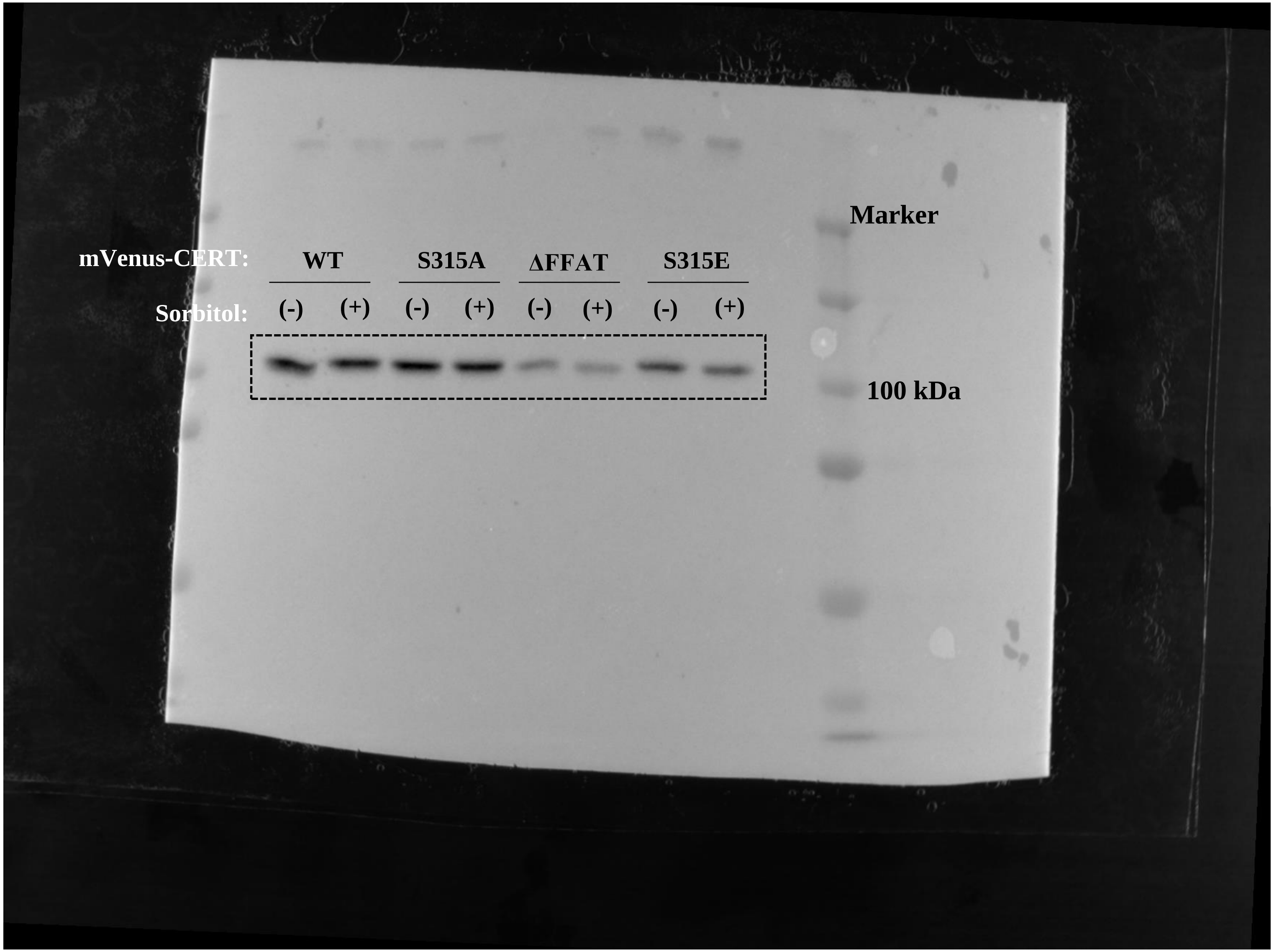

Figure S3B

WB: VAP-A

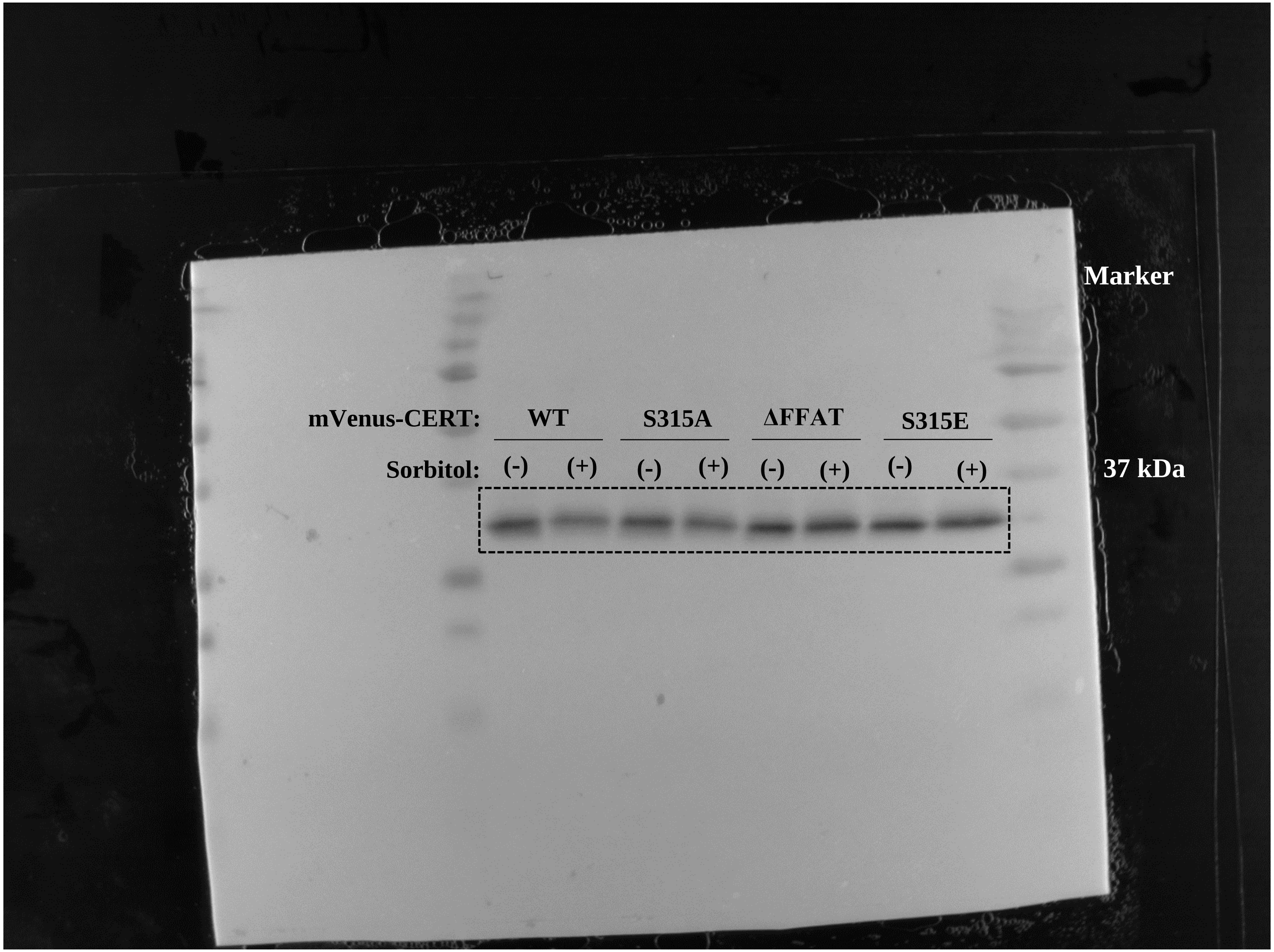

Figure S3B

WB: GAPDH

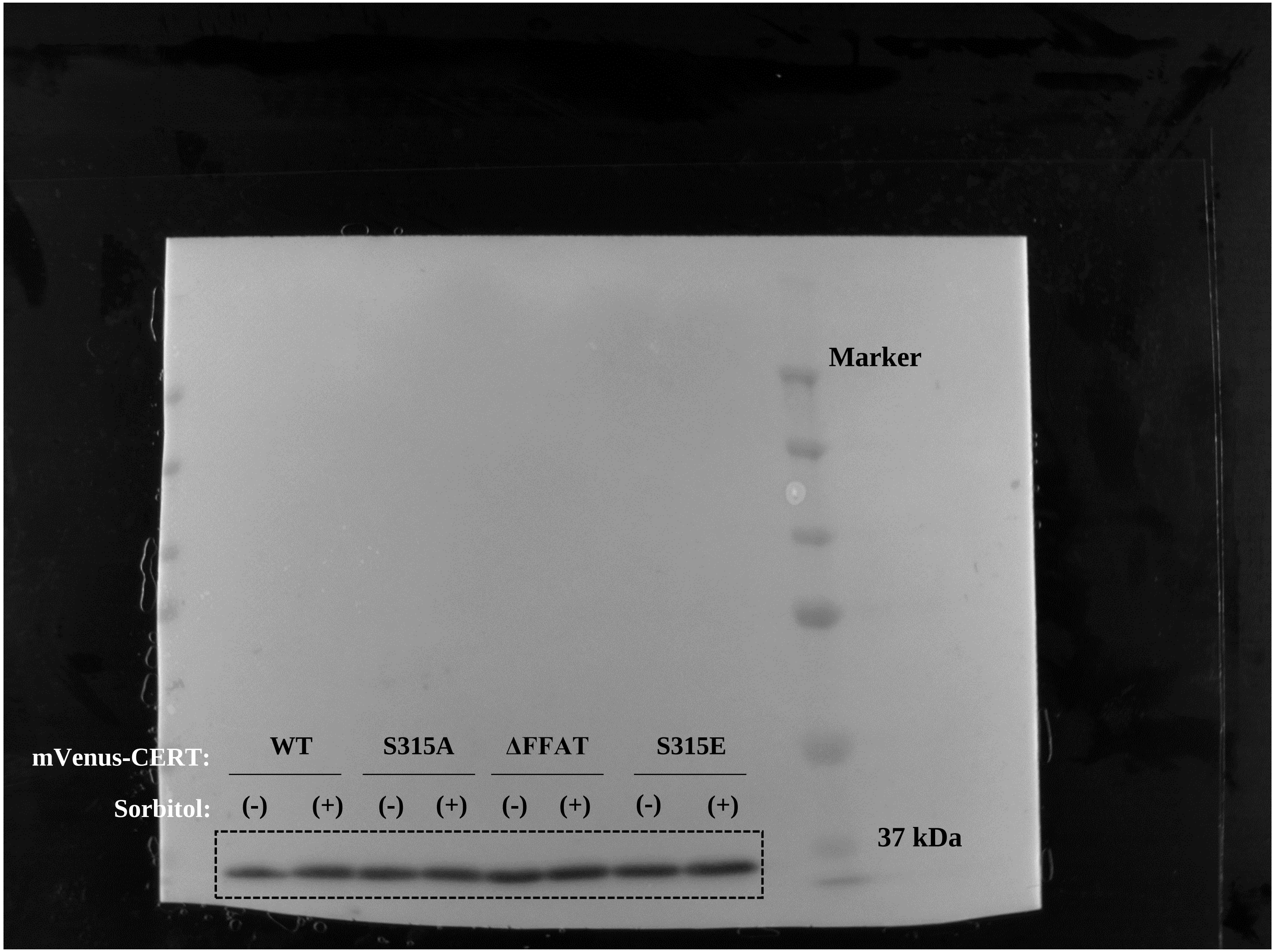

Figure S4A

TLC

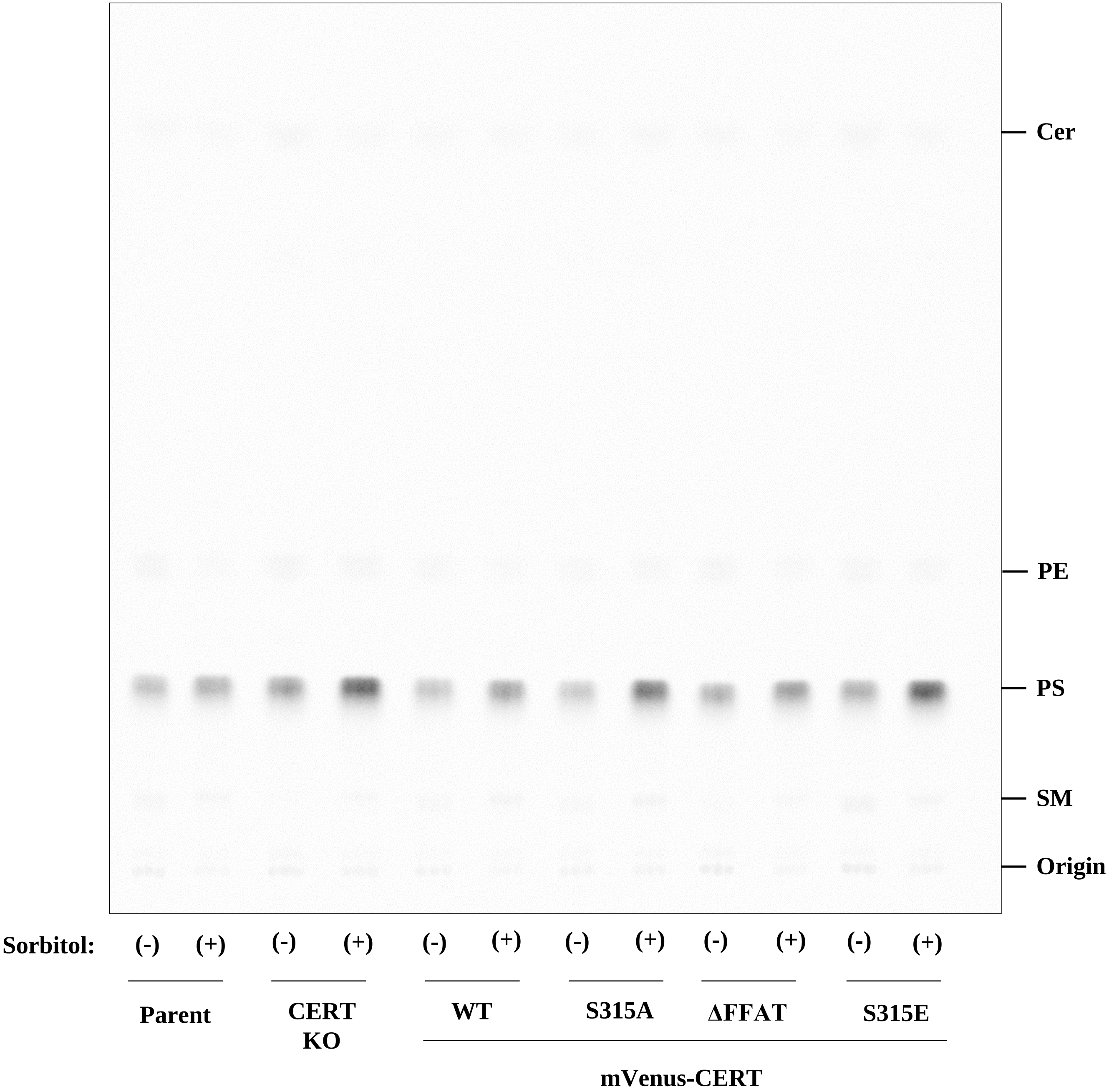

Figure S4B

TLC

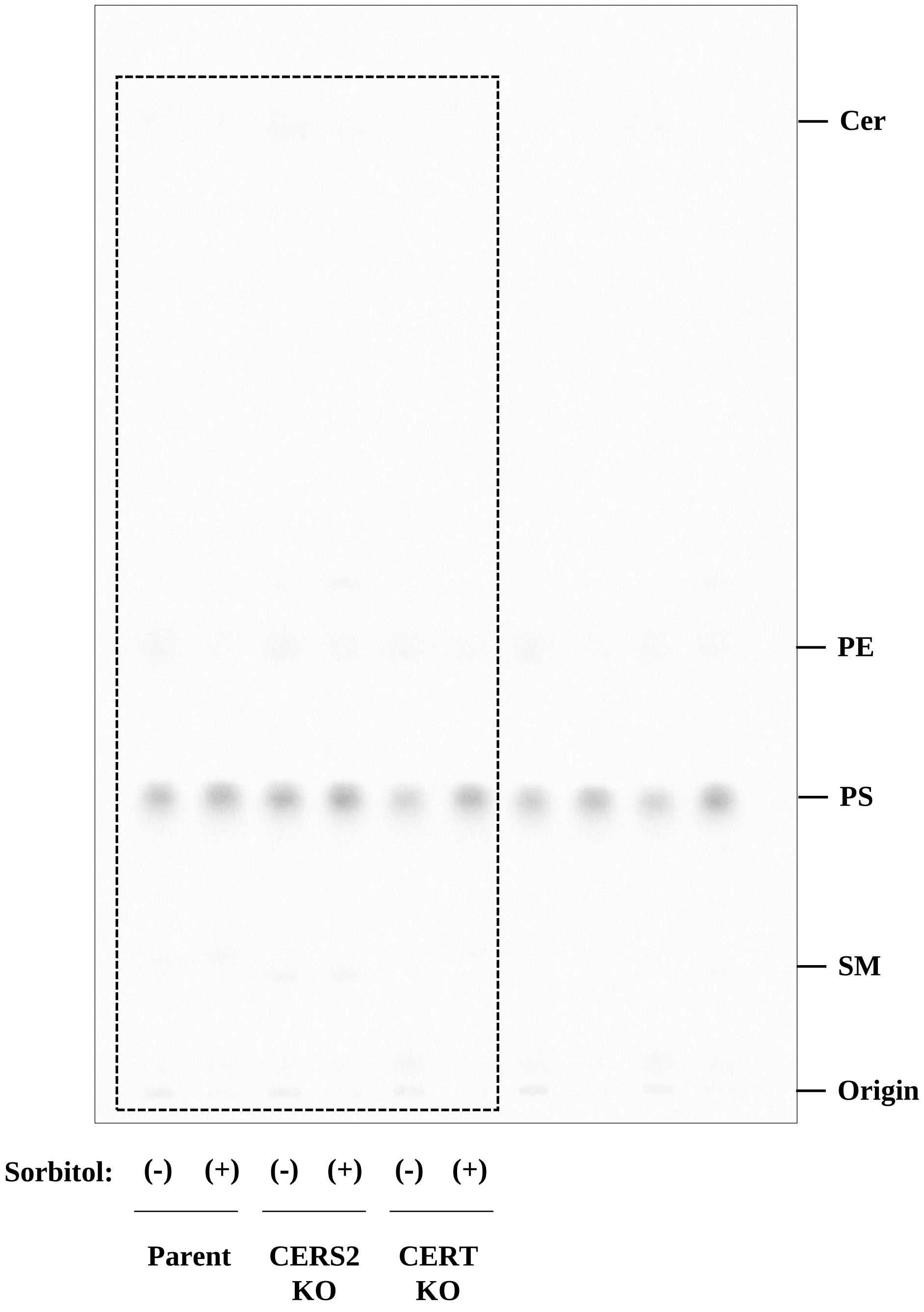

Figure S5A

TLC

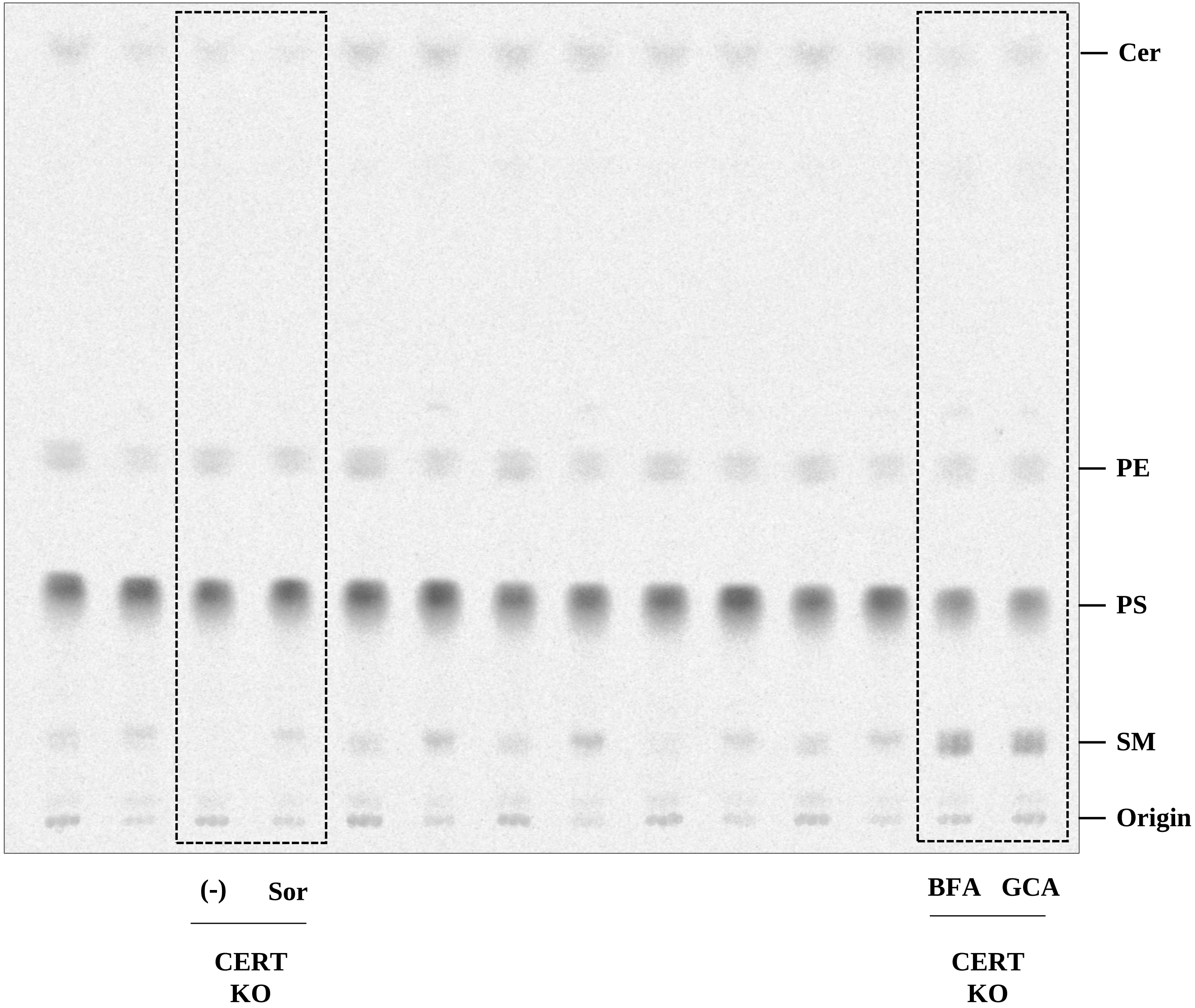

Figure S5B

TLC

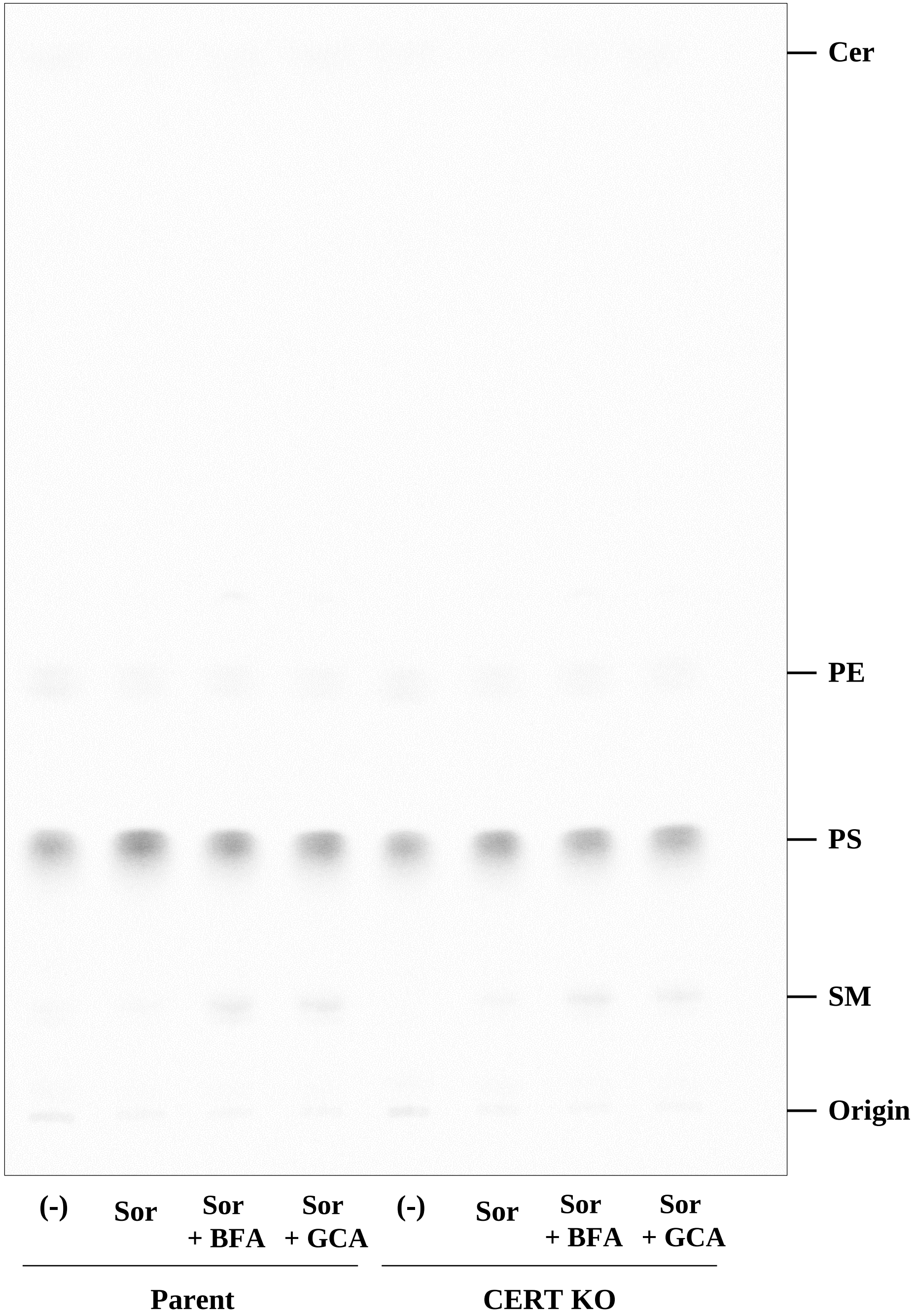

Supplement: Supplementary file 1 [file ijms-23-04025-s001.zip › ijms-1504012-supplementary.pdf]
